# Conjugation Chemistry Markedly Impacts Toxicity and Biodistribution of Targeted Nanoparticles, Mediated by Complement Activation

**DOI:** 10.1002/adma.202409945

**Published:** 2024-12-11

**Authors:** Michael H. Zaleski, Liam S. Chase, Elizabeth D. Hood, Zhicheng Wang, Jia Nong, Carolann L. Espy, Marco E. Zamora, Jichuan Wu, Lianne J. Morrell, Vladimir R. Muzykantov, Jacob W. Myerson, Jacob S. Brenner

**Affiliations:** ^1^ Department of Systems Pharmacology and Translational Therapeutics The Perelman School of Medicine University of Pennsylvania 421 Curie Blvd., 354 BRB II/III Philadelphia PA 19104 USA; ^2^ Department of Medicine University of Pennsylvania 3400 Civic Center Boulevard Philadelphia PA 19104 USA

**Keywords:** complement system, conjugation chemistry, drug delivery, nanomedicine, targeted nanoparticles

## Abstract

Conjugation chemistries are a major enabling technology for the development of drug delivery systems, from antibody‐drug conjugates to antibody‐targeted lipid nanoparticles inspired by the success of the COVID‐19 vaccine. However, here it is shown that for antibody‐targeted nanoparticles, the most popular conjugation chemistries directly participate in the activation of the complement cascade of plasma proteins. Their activation of complement leads to large changes in the biodistribution of nanoparticles (up to 140‐fold increased uptake into phagocytes of the lungs) and multiple toxicities, including a 50% drop in platelet count. It is founded that the mechanism of complement activation varies dramatically between different conjugation chemistries. Dibenzocyclooctyne, a commonly used click‐chemistry, caused aggregation of conjugated antibodies, but only on the surface of nanoparticles (not in bulk solution). By contrast, thiol‐maleimide chemistry do not activate complement via its effects on antibodies, but rather because free maleimide bonded to albumin in plasma, and clustered albumin is then attacked by complement. Using these mechanistic insights, solutions are engineered that reduced the activation of complement for each class of conjugation chemistry. These results highlight that while conjugation chemistry is essential for the future of nanomedicine, it is not innocuous and must be designed with opsonins like complement in mind.

## Graphical abstract

Conjugation chemistries are a critical technology in nanomedicine, enabling precise functionalization of nanoparticle surfaces (e.g., via attachment of targeting moieties). This work investigates how conjugation chemistry impacts nanoparticle‐induced complement activation. The results demonstrate that modern conjugation chemistries, although bio‐orthogonal, are not innocuous and must be chosen with opsonins like complement in mind.

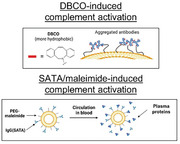

## 1 Introduction

The 2022 Nobel Prize in Chemistry was awarded for inroads in conjugation chemistries, highlighting the importance of these ligation methods for a broad range of biomedical applications. These chemistries allow for precise functionalization of nanoparticle surfaces, such as the attachment of targeting moieties, to facilitate targeted delivery of therapeutics. Conjugation chemistry is a key enabling technology for several nanomedicines and drug delivery systems (DDSs) in preclinical and clinical development, such as targeted nanoparticles and antibody drug conjugates (ADCs).^[^ ^1^, ^2^ ^]^

A key achievement for the field was the development of highly specific and seemingly innocuous conjugation chemistries. Indeed, modern conjugation chemistries are benign from a purely chemical perspective – they are highly specific, do not require toxic solvents or catalysts, and can be performed under mild conditions (e.g., neutral pH, ambient temperature).^[^ ^3^, ^4^, ^5^ ^]^ Despite their impressive properties, these conjugation chemistries may not be completely innocuous from a biologic perspective. This has been investigated for ADCs, in which the choice of chemical linker can impact thermal stability, antigen affinity, and ultimately pharmacokinetics and therapeutic efficacy.^[^ ^6^, ^7^, ^8^ ^]^ Understanding the impact of conjugation chemistry on ADC performance enabled the successful translation of ADCs to clinical use, underscoring the importance of studying conjugation chemistry during the development of new DDSs.^[^ ^9^, ^10^ ^]^

One emerging class of nanomedicines is antibody‐nanoparticle conjugates, which consist of a monoclonal antibody (or fragment thereof) as the targeting moiety and a nanoparticle carrying a therapeutic payload. At least 10 clinical trials with antibody (or antibody‐derivative)‐nanoparticle conjugates have been conducted in the past 10 years.^[^ ^11^ ^]^ Many more such antibody‐targeted nanoparticles are now in development as part of the RNA‐lipid‐nanoparticle (LNP) boom catalyzed by the success of the COVID‐19 vaccines. Yet, relatively little is known about the impact of conjugation chemistry on the performance of antibody‐targeted nanoparticles. Numerous conjugation chemistries have been developed for the attachment of antibodies to nanoparticle surfaces.^[^ ^12^, ^13^ ^]^ A small body of research compares covalent and non‐covalent conjugation strategies for gold nanoparticles and their impact to sensing applications.^[^ ^14^, ^15^ ^]^ But in total, limited knowledge exists regarding the impact of conjugation chemistry on the in vivo behavior of functionalized nanoparticles.

In this work, we show that modern conjugation chemistries have surprising and large‐magnitude consequences for toxicity and biodistribution of antibody‐targeted nanoparticles. Our mechanistic studies show that conjugation chemistries’ negative effects are primarily mediated by the complement cascade of plasma proteins. Complement is a set of ∼40 proteins that rapidly bind to the surface of microbes invading the blood.^[^ ^16^ ^]^ While complement evolved to attack microbes, it can do the same to nanomedicines,^[^ ^17^, ^18^, ^19^ ^]^ and here we show that the choice of conjugation chemistry greatly impacts complement activation.

Numerous conjugation chemistries have been used for constructing drug delivery systems, such as copper‐catalyzed or copper free click chemistry, oxime, N‐hydroxysuccinimide (NHS), and thiol‐maleimide chemistries.^[^ ^20^ ^]^ In this work, we focus on two of the most widely used conjugation chemistries: DBCO (dibenzocyclooctyne)‐azide and thiol‐maleimide. DBCO‐azide is a strain‐promoted azide‐alkyne cycloaddition (SPAAC), a widely used bio‐orthogonal click chemistry. It has recently become one of the most frequently utilized chemistries for preclinical studies, due to its relative ease of use, specificity, “bio‐orthogonality” and high yield.^[^ ^5^, ^21^ ^]^ Thiol‐maleimide is the most common chemistry for ADCs in clinical use.^[^ ^22^ ^]^ Additionally, of the few antibody‐nanoparticle conjugates tested in clinical trials,^[^ ^11^ ^]^ the majority use thiol‐maleimide chemistry.^[^ ^23^, ^24^, ^25^ ^]^

First, we explored the impact of conjugation chemistry on biodistribution, showing that the choice of conjugation chemistry dramatically affects lung uptake in mice with acute inflammation induced by intravenous bacterial lipopolysaccharide (LPS). Experiments with knockout mice show the lung uptake is almost entirely due to complement activation. Adverse effects of complement activation are seen in both LPS‐injured and healthy mice. Delving deeper into this mechanism, we discover that DBCO‐azide chemistry leads to protein aggregation on the nanoparticle's surface, due to the hydrophobicity of the DBCO moiety. Previous literature has shown that protein aggregates correlate with complement activation,^[^ ^26^, ^27^ ^]^ and here we find that nanoparticles with proteins aggregated on their surface are prone to complement‐mediated changes in biodistribution. We show that thiol‐maleimide chemistry results in non‐specific conjugation of the maleimide group to thiol‐containing albumin in the blood and subsequent complement activation. This result is in agreement with previous studies showing the potential cross‐reactivity of maleimide with biological molecules in vivo.^[^ ^8^ ^]^ Armed with this knowledge, we engineered solutions that minimize complement activation caused by the nanoparticles’ particular choice of conjugation chemistry.

## 2 Results and Discussion

### 2.1 Production and Characterization of Antibody‐Liposome Conjugates

We chose antibody‐liposome conjugates as a model nanoparticle to investigate the effects of conjugation chemistry. Liposomes were chosen because they compose the plurality of clinically approved nanoparticle products.^[^ ^11^ ^]^ For the antibody, we chose non‐specific IgG, as it will allow the results to be generalizable and not limited to a particular antibody clone or target determinant.

**Figure 1:**
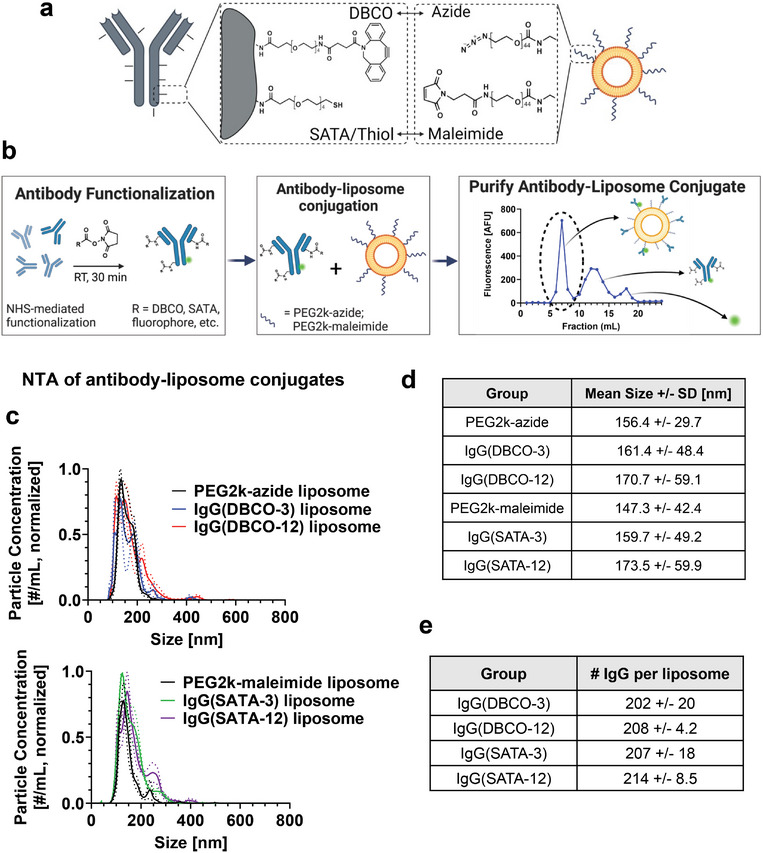
Production and characterization of antibody‐liposome conjugates. (a) Schematic for the two primary conjugation chemistries in this study: DBCO‐azide (a copper‐free click chemistry) and SATA/thiol‐maleimide. (b) Method for producing antibody‐nanoparticle conjugates. Antibodies were modified with a reactive moiety (DBCO or SATA) and fluorescent tag via NHS ester addition. Modified antibodies were conjugated to liposomes containing the appropriate reactive lipid (azide or maleimide). Size‐exclusion chromatography (SEC) was used to purify antibody‐liposome conjugates. The fluorescent tag was used to quantify the antibody‐liposome conjugation efficiency by comparing fluorescent signal in fractions corresponding to unconjugated antibody with fluorescent signal in fractions corresponding to liposomes. Fractions containing antibody‐liposome conjugates were used for subsequent studies. (c) Nanoparticle Tracking Analysis (NTA) was used to measure the size distribution of antibody‐liposome conjugates. Solid lines indicate the mean, and dotted lines indicate standard error of the mean (SEM) (*n* = 3 batches per condition). Note the similar size distribution for all conjugation chemistries. (d) Mean size of antibody‐liposomes conjugates, determined from NTA (*n* = 3 batches per condition). Conjugation of IgG increases the liposome size by 10 to 25 nm, as expected due to the 10 nm size of IgG. Note the similar size of DBCO‐azide and SATA‐maleimide conjugation chemistries. (e) Calculated number of IgG molecules per liposome for each conjugation chemistry (See Figures S3 and S4 (Supporting Information) for detailed methods and calculations). 200 IgG molecules per liposome was the target reaction yield, and particles were only used if the actual number of IgG per liposome was within 15% of the target (170 to 230 IgG per liposome).

IgG was conjugated to liposomes using either DBCO‐azide or thiol‐maleimide chemistries, two of the most widely‐used conjugation chemistries (**Figure** **1**a). First, IgG is modified with DBCO or SATA (S‐acetyl thioacetate). The SATA group is subsequently deprotected to a thiol, and herein we will refer to this chemistry as SATA‐maleimide. IgG is then conjugated to liposomes containing the respective reactive group (azide or maleimide) on the terminal end of a PEGylated lipid. After conjugation, the mixture is purified using size‐exclusion chromatography (SEC) to both quantify the conjugation efficiency of IgG to liposomes as well as to purify IgG‐liposome conjugates from unconjugated antibody (Figure 1b). We chose to modify IgG with either 3 or 12 reactive groups per IgG molecule (designated IgG(DBCO‐3), IgG(DBCO‐12), IgG(SATA‐3), and IgG(SATA‐12)). Although 3 reactive groups per IgG is sufficient for robust conjugation, we included a condition with increased modification to increase the signal‐to‐noise when measuring effects of the different conjugation chemistries. Including conditions with high numbers of DBCO and SATA allowed us to probe how the reactive groups themselves impact performance (e.g., biodistribution, toxicities) of antibody‐liposome conjugates.

We performed various quality control measurements to ensure that all antibody‐nanoparticle conjugates were substantially similar, with conjugation chemistry being the only major variable. Dynamic Light Scattering (DLS) of IgG (prior to conjugation to liposomes) indicates minimal levels of IgG aggregation due to modification with DBCO and SATA (Figure S1, Supporting Information). UV spectroscopy was used to confirm the number of reactive groups added per IgG (Figure S2, Supporting Information). Nanoparticle Tracking Analysis (NTA) was performed to characterize the size distribution of liposomes before and after IgG conjugation (Figure 1). The size profiles are largely homogeneous, with similar profiles for all conjugation chemistries. Quantification of mean size from NTA data shows that all groups are similar in size, ranging from 145  to 175 nm (Figure 1d). The size of liposomes increases by 10 to 20 nm after conjugation to IgG, as expected given that IgG molecules have an approximate diameter of 10 nm. DLS analysis of the antibody‐liposome conjugates showed a similar trend in sizes as NTA (Figure S7, Supporting Information). Conjugation reactions were conducted to yield 200 IgG per liposome. The actual number of IgG per liposome was within 15% of this target (Figure 1e). See Figures S3–S6 (Supporting Information) for detailed methods and calculations regarding the number of IgG per liposome.

Finally, we analyzed the percentage of aggregated particles from NTA data (Figure S6b, Supporting Information). We defined aggregated liposomes as the fraction of particles over 300 nm. The size cutoff was chosen as double the size of PEGylated liposomes with no IgG conjugated (150 nm x 2 for PEG‐azide and PEG‐maleimide liposomes). All preparations contained less than 4% aggregates. Increasing the modification of IgG (e.g., IgG(SATA‐12) versus IgG(SATA‐3)) does increase the percentage of aggregated liposomes. However, the percentage of aggregated liposomes was nearly identical when comparing SATA‐maleimide and DBCO‐azide chemistries. In sum, the quality control measurements confirm that antibody‐liposome conjugates are substantially similar for all conjugation chemistries, with the chemistry itself being the primary variable.

### 2.2 Conjugation Chemistry Affects the Biodistribution and Toxicity of Antibody‐Liposome Conjugates, Due to Complement Activation, Especially in the Setting of Background Inflammation

**Figure 2:**
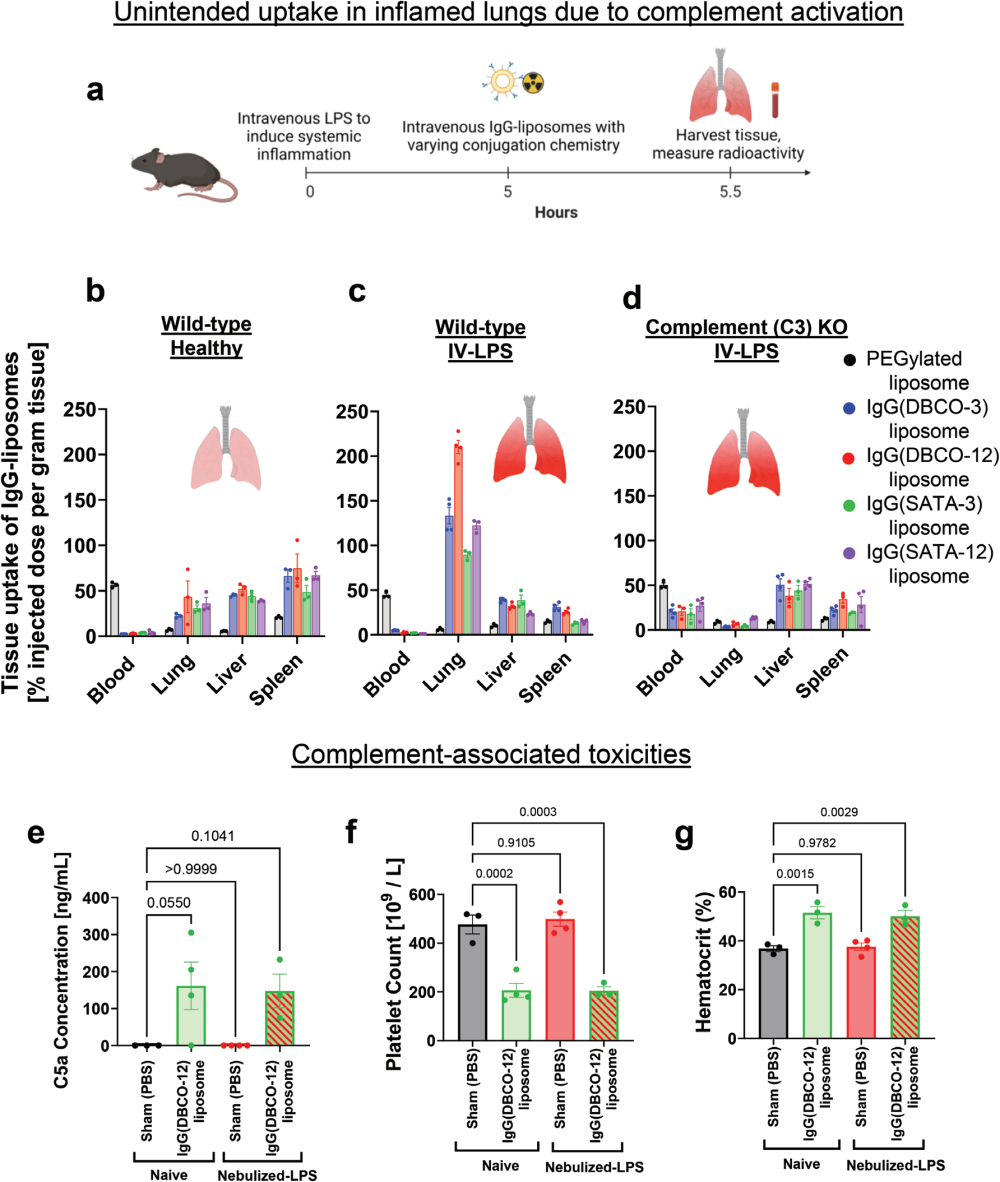
Conjugation chemistry affects the biodistribution and toxicity of antibody‐liposome conjugates, due to complement activation, especially in the setting of background inflammation. (a) The impact of conjugation chemistry on the biodistribution of antibody‐liposome conjugates was investigated in mice. Non‐specific IgG was modified with a 3‐fold or 12‐fold molar excess of DBCO (IgG(DBCO‐3); IgG(DBCO‐12)), or SATA (IgG(SATA‐3), IgG(SATA‐12)), then conjugated to liposomes. IgG‐liposome conjugates were injected intravenously into mice and biodistribution was measured via radioactivity. PEGylated liposomes (with no IgG conjugated to the surface) were included as a control. (b) In healthy mice, IgG‐liposomes primarily distributed to the liver and spleen, the primary clearance organs. The distribution did not change for different conjugation chemistries. (c) However, in mice with systemic, acute inflammation induced by intravenous‐LPS, liposomes had a 4‐fold increase in lung accumulation, which was further influenced by conjugation chemistry. Increased modification of IgG with DBCO significantly increased lung uptake. (d) Complement activation is necessary for lung uptake, as demonstrated by complete abrogation of lung uptake in mice with knockout of complement protein C3 (C3‐KO). For (b‐d), *n* = 3‐4 mice per group. (e–g) IgG‐liposomes cause complement activation and associated toxicities in vivo. Mice were exposed to nebulized‐LPS then treated with IgG‐liposome conjugates, and whole blood was collected 10 min later. (e) The concentration of C5a, a direct product of complement activation, dramatically increased after liposome administration. (f,g) A significant decrease in platelet count and increase in hematocrit, toxicities associated with complement activation, also occurred. For (e‐g), comparisons made by ordinary one‐way ANOVA with Dunnett's multiple comparison test, with “Naïve, Sham (PBS) treatment” as the control (*n* = 3–4 mice per group).

We began by studying the impact of conjugation chemistry on the in vivo performance of antibody‐nanoparticle conjugates. In vivo studies allow us to probe the many complex and dynamic systems that impact nanoparticle behavior, such as the immune system, red blood cells, protein corona formation, and pathology. We first investigated nanoparticle biodistribution because it is a fundamental metric of nanomedicine performance and translatability. We produced antibody‐liposome conjugates as described in Figure 1, with a tracer amount of I‐125 labeled IgG included to facilitate precise and quantifiable organ distribution data. We IV‐injected IgG‐liposome conjugates into mice and measured the organ distribution via radioactivity. Liposomes with no IgG conjugated to the surface (PEGylated liposomes) were included as a control (**Figure** **2**a; Figure S8, Supporting Information).

In healthy mice, the majority of the dose of IgG‐liposomes accumulated in the liver and spleen (the primary clearance organs), and the biodistribution did not vary for different conjugation chemistries (Figure 2b). PEGylated liposomes primarily remained in circulation in the blood. However, in mice with acute inflammation induced by intravenous LPS, IgG‐liposomes primarily accumulated in the lungs (Figure 2c). Additionally, the extent of lung accumulation was strongly influenced by conjugation chemistry. IgG(DBCO‐12) liposomes accumulated in the lungs at a concentration of 210% injected dose per gram of tissue (%ID/g), compared to only 133%ID/g for IgG(DBCO‐3) liposomes. For SATA‐maleimide chemistry, the extent of IgG modification with SATA had a smaller impact on lung accumulation (122%ID/g for IgG(SATA‐12) liposomes versus 90%ID/g for IgG(SATA‐3) liposomes). Importantly, these liposomes are *not* targeted to the lungs, since the IgG used does not specifically bind to any epitope present in the lungs. The observed lung uptake is unintended and could actually interfere with designed targeting mechanisms of antibody‐nanoparticle conjugates. Since this phenomenon is only seen in inflamed mice, it is likely missed during preclinical pharmacokinetic studies, which are typically performed in healthy animals. In the inflamed IV‐LPS mice, the control PEGylated liposomes still primarily remained in circulation in the blood.

We next sought to understand the cause of the observed lung uptake. Our previous studies suggest that uptake of nanoparticles in the lung can in some circumstances be due to complement activation, followed by recognition of complement‐bound nanoparticles by marginated neutrophils that dwell in the lungs’ capillaries.^[^ ^28^, ^29^ ^]^ To confirm the involvement of complement, we performed a biodistribution study in mice with knockout of complement protein C3 (C3‐KO). C3 is a critical junction of the complement cascade, with all complement pathways (classical, alternative, lectin) depending on C3. Lung accumulation of nanoparticles was completely eliminated in C3‐KO mice, demonstrating that complement is necessary for the lung uptake phenomena observed in acute inflammation (Figure 2d). This dramatic decrease in lung uptake occurred for all conjugation chemistries, with all conditions exhibiting less than 15%ID/g in the lungs. Together, the results in Figure 2b–d suggest that conjugation chemistry plays a significant role in the activation of complement by nanoparticles. This was not previously known nor suspected, as none of the three complement pathways are directly activated by the reactive moieties involved in bioconjugate chemistry.

To confirm that accuracy of I‐125 tracing of IgG, control biodistribution studies were performed using a membrane marker to trace the liposome itself. The liposome membrane was traced by chelating indium‐111 (In‐111) to chelator‐containing lipids (DSPE‐DTPA) incorporated into the liposome formulation. Results using both In‐111 membrane tracer and I‐125 antibody tracer yielded similar results (Figure S9, Supporting Information). For both tracers, we observed a significant increase in lung uptake in mice given IV‐LPS compared to naïve mice, and a complete elimination of lung uptake in C3‐KO mice. Control biodistributions were performed on the liposome alone (PEGylated, with no IgG conjugated to the surface, as shown in Figure 2b–d) and IgG alone (not conjugated to liposomes). Both liposomes and IgG stay in circulation for 30 min, with the majority of the dose in blood and minimal distribution to tissue (Figure S10, Supporting Information). Neither liposomes alone nor IgG alone show evidence of lung uptake due to complement activation.

To understand the biodistribution of antibody‐liposome conjugates on a cellular level, we performed flow cytometry studies to assess the cell types responsible for complement‐dependent lung uptake. Fluorescent liposomes were administered to mice and the lungs were analyzed via flow cytometry to determine the cell types taking up IgG‐liposome conjugates (Figure S11, Supporting Information). Neutrophils were the main cell type responsible for liposome accumulation in the lungs. 97% of neutrophils were liposome positive and neutrophils exhibited the highest mean fluorescence intensity (MFI) of any cell type. Neutrophil uptake was completely eliminated in C3‐KO mice, confirming the necessity of complement in neutrophil‐mediated lung uptake. These results align with previous studies demonstrating that nanoparticle uptake by neutrophils is complement dependent.^[^ ^29^ ^]^

Given that antibody properties, such as subclass and glycosylation, are known to modulate complement activation, we assessed the generalizability of complement‐mediated lung uptake by performing biodistributions of IgG‐liposomes made with monoclonal mouse IgG of different subclasses (Figure S12, Supporting Information). We tested subclass IgG1 and IgG2a. Out of the four subclasses of murine IgG (1, 2a, 2b, and 3), IgG1 exhibits the least amount of complement fixation.^[^ ^30^ ^]^ The IgG2a subclass exhibits a high degree of complement fixation, comparable to that of IgG2b and IgG3 subclasses.^[^ ^30^ ^]^ Therefore, the use of IgG1 and IgG2a brackets the full range of complement fixation by murine IgG subclasses. The trends in lung uptake when using mouse IgG (Figure S12, Supporting Information) match the results from studies using rat IgG (Figure 2b,d). Namely, increased modification with DBCO caused increased lung uptake for both subclasses of IgG. Also, IgG2a accumulated in the lungs significantly more than IgG1, in agreement with literature that IgG2a fixes complement more strongly than IgG1.

Having found that complement binding greatly affects targeted nanoparticles' biodistribution, we next asked whether this activation of complement leads to toxicity. To generalize our results beyond the first model of systemic inflammation (IV‐LPS), here we induced local inflammation using nebulized‐LPS. Into nebulized‐LPS mice, we IV‐injected IgG(DBCO‐12) liposomes and collected blood 10 min later for measurement of acute complement toxicities. We measured the plasma concentration of C5a, a direct product of complement activation and a source of many anaphylaxis‐like toxicities.^[^ ^31^ ^]^ C5a was undetectable in mice administered a sham treatment, but increased to >100 ng mL^−1^ in mice administered IgG‐liposome conjugates (Figure 2e). Additionally, we observed a 50% decrease in platelet count and 40% increase in hematocrit (Figure 2f,g). An acute drop in platelet count and an increase in hematocrit are known to correlate with complement activation in multiple species.^[^ ^32^, ^33^, ^34^ ^]^ Both are serious side‐effects, with low platelet count indicating dysregulated coagulation and the potential for severe bleeding, and elevated hematocrit likely due to complement‐mediated increases in capillary permeability that causes plasma to leak from the blood into the tissues. All complement‐associated toxicities occurred regardless of inflammation status, suggesting they are a ubiquitous concern for antibody‐liposome conjugates. Therefore, we next strove to understand the mechanisms underlying how conjugation chemistry impacts complement activation, as this could ultimately lead to rational design of antibody‐nanoparticle conjugates to minimize these toxicities.

### 2.3 In Vitro Complement Activation Profiles Suggest the Disparate Mechanisms by which DBCO‐Azide and SATA‐Maleimide Induce Complement Activation

Having identified complement as a key system influenced by conjugation chemistry, we next sought to understand in more detail how different conjugation chemistries influence complement activation. We performed in vitro analyses of complement activation for both DBCO‐azide and SATA/thiol‐maleimide chemistries. We produced IgG‐liposome conjugates with varying conjugation chemistries, as described in Figure 1 and tested in Figure 2b–d. In addition, we varied the number of IgG molecules per liposome, ranging from 6 to 200 IgG per liposome. Previously, we held the IgG density constant at 200 IgG per liposome in order to isolate the effects of conjugation chemistry. Now, we sought to investigate the impact of both conjugation chemistry and antibody density on complement activation.

**Figure 3:**
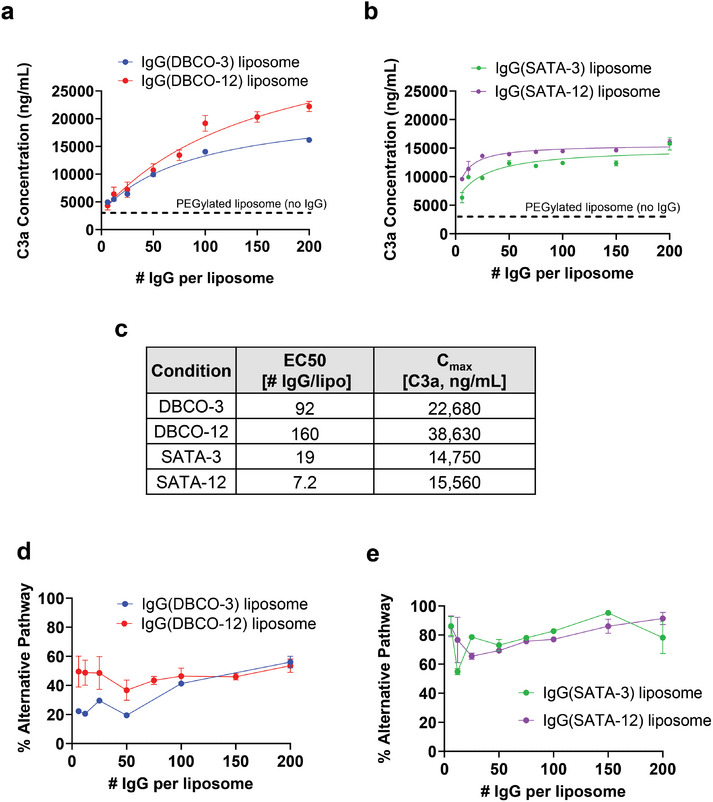
In vitro complement activation profiles further elucidate the disparate mechanisms by which DBCO‐azide and SATA‐maleimide induce complement activation. IgG‐liposome conjugates, with varying amounts of IgG per liposome, were incubated with serum, and C3a concentration was measured via ELISA. Studies were performed for both DBCO (a) and SATA (b) chemistries (*n* = 2–4 replicates per condition). The data were fit to the Hill Equation and best‐fit parameter values are presented (c). Increased modification of IgG with DBCO (12 vs 3 DBCO per IgG) leads to significantly higher complement activation, as seen in the higher estimate of C_max_ for IgG(DBCO‐12) (a,c). This observation suggests that DBCO‐azide induced complement activation is driven by DBCO moiety. However, increased modification of IgG with SATA does not significantly change the complement activation profile nor the estimate for C_max_ (b,c), indicating that SATA‐maleimide induced complement activation is driven by residual free maleimide groups, not the SATA modification. For SATA‐maleimide, maximum complement activation is reached even at low IgG densities, as demonstrated by the low EC50 values. This suggests the main driver of complement activation is free maleimide reactive groups on the surface of the liposome, as opposed to the SATA moiety or IgG conjugated to the liposome. (d,e) The complement activation assay used in (a,b) was performed in the presence of Gelatin Veronal Buffer (GVB) to inhibit the classical pathway of complement activation (*n* = 2–4 replicates per condition). C3a concentration in the presence of GVB was divided by C3a concentration without GVB to calculate the percent of complement activation due to the alternative pathway. For SATA‐maleimide chemistry, the majority of complement activation (≈80%) is due to the alternative pathway. This is similar for all IgG densities tested (6 to 200 IgG per liposome) and for both SATA‐3 and SATA‐12 chemistries, again suggesting that the maleimide group, not SATA/thiol, drives complement activation. DBCO‐azide chemistry has a larger contribution from the classical pathway compared to SATA‐maleimide. Increased modification with DBCO leads to an increased contribution from the alternative pathway, but only for low IgG densities.

To quantify complement activation caused by IgG‐liposome conjugates, we incubated the particles in mouse serum in vitro, then quenched the complement reaction with EDTA after 10 min. Complement activation was assessed by measuring the concentration of C3a, a direct product of complement activation, via ELISA. The pattern of complement activation appears markedly different between DBCO‐azide and SATA‐maleimide chemistries (**Figure** **3**a,b). To quantify the shape of each curve, we fit the data to the Hill Equation and estimated the parameters EC50, representing the density of IgG per liposome required to induce half‐maximal complement activation, and C_max_, the maximum concentration of C3a (Figure 3c).

For DBCO‐azide chemistry, increased modification of IgG with DBCO results in greater complement activation. The C_max_ for IgG(DBCO‐12) liposomes is 38630 ng mL^−1^ compared to only 22680 ng mL^−1^ for IgG(DBCO‐3) liposomes. In contrast, increased modification of IgG with SATA causes only a slight increase in C_max_ (14750 to 15560 ng mL^−1^ for IgG(SATA‐3) and IgG(SATA‐12) liposomes, respectively). These results mirror the outcome of our initial biodistribution study, where increased DBCO modification led to a 58% increase in lung uptake, but increased SATA modification only led to a 37% increase in lung uptake (Figure 1d).

For DBCO‐azide chemistry, complement activation appears to be a strong function of IgG density. C3a concentration continuously increases as IgG density increases from 6 to 200 IgG per liposome. However, for SATA‐maleimide chemistry, complement activation is less dependent on IgG density. This observation can be quantified by the EC50 values for each group. IgG(SATA‐3) and IgG(SATA‐12) liposomes exhibit low EC50 values (19 and 7 IgG per liposome, respectively), indicating that maximum complement activation is reached even at low IgG densities. The EC50 values for IgG(DBCO‐3) and IgG(DBCO‐12) liposomes are much greater (92 and 160 IgG per liposome, respectively) indicating that IgG density continues to influence complement activation up to≈200 IgG per liposome.

We next sought to assess the contribution of the complement activation pathways (classical, alternative, lectin) to overall complement activation caused by IgG‐liposome conjugated. The *in* vitro complement activation assay described above was performed with Gelatin Veronal Buffer (GVB) added to serum prior to liposome addition. GVB inhibits the classical pathway and therefore allows isolation of the alternative pathway. The percent of complement activation due to the alternative pathway was calculated as C3a concentration in the presence of GVB divided by C3a concentration without GVB (Figure 3d,e). For SATA‐maleimide chemistry, the majority of complement activation (≈80%) is due to the alternative pathway. This is true for all IgG densities tested (6 to 200 IgG per liposome). DBCO‐azide chemistry has a larger contribution from the classical pathway compared to SATA‐maleimide. For IgG(DBCO‐3) chemistry, the classical pathway constitutes the majority of complement activation (≈80%) at low IgG densities (<50 IgG per liposome). At higher IgG densities, the contribution from the alternative pathway actually increases and there is roughly equal contribution from each pathway. For IgG(DBCO‐12), there is roughly equal contribution from the alternative and classical pathways for all IgG densities.

Together, these patterns may provide insights into the mechanisms of complement activation. For DBCO‐azide chemistry, the impact of DBCO modification on complement activation and the higher contribution from the classical pathway suggest that the DBCO moiety and IgG molecules are the primary drivers of complement activation. However, for SATA‐maleimide chemistry, the SATA moiety and IgG molecules have a minimal impact on complement activation. Additionally, complement activation is largely driven by the alternative pathway, suggesting that a different component of the system, other than IgG is the main cause of complement activation when using SATA‐maleimide chemistry.

Given that antibody structure and glycosylation are known to modulate complement activation, we sought to expand the generalizability of the previous observations by testing multiple IgG with differing interactions with complement. As described in the text for Figure 2, we used murine IgG of subclasses IgG1 and IgG2a, as these bracket the full range of complement fixation by murine IgG subclasses. We analyzed the in vitro complement activation of murine IgG‐liposome conjugates, and observed similar patterns as in Figure 3 (Figure S15, Supporting Information). Namely, increased modification of IgG with DBCO leads to significantly higher complement activation. For SATA‐maleimide chemistry, increased modification with SATA has little impact on complement activation, and maximum complement activation is reached even at low IgG densities.

### 2.4 DBCO‐Azide Conjugation Chemistry Induces Complement Activation via Protein Aggregation on Nanoparticle Surfaces

**Figure 4:**
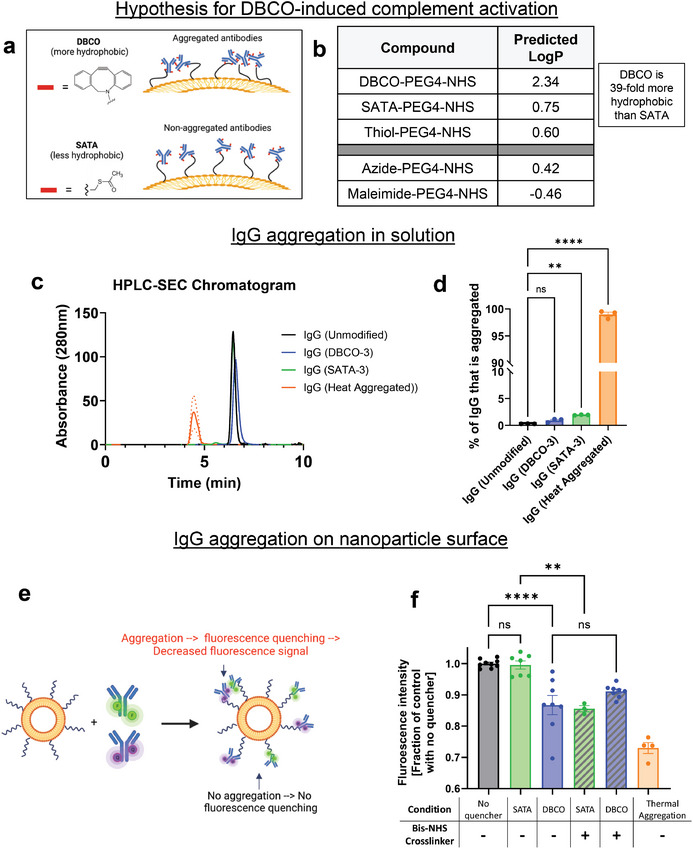
DBCO‐azide conjugation chemistry induces complement activation via protein aggregation on nanoparticle surfaces. (a) Schematic depicting our hypothesis that DBCO‐modified IgG exhibits more pronounced aggregation compared to SATA‐modified IgG, due to the greater hydrophobicity of DBCO moiety. (b) DBCO is 32‐fold more hydrophobic than SATA, per model predicted logP. (c,d) The aggregation of modified IgG in solution (not conjugated to a liposome) was assessed using HPLC‐SEC (*n* = 3 replicates per condition). (c) The chromatograms show a similar elution profile for all conditions (unmodified, DBCO‐modified, and SATA‐modified IgG). As a positive control, IgG was heat‐treated to cause aggregation, resulting in earlier elution time. (d) The bar graph quantifies the percent of IgG aggregated in each condition. All conditions have <2% aggregates, indicating that DBCO and SATA modification alone do not induce IgG aggregation. Comparisons made by ordinary one‐way ANOVA with Dunnett's multiple comparison test, with unmodified IgG as the control. (e) IgG aggregation on the liposome surface was measured utilizing fluorescence quenching. When aggregation occurs, the fluorescent and quencher molecules come within close proximity, resulting in a decrease in fluorescence intensity. (f) DBCO‐modified IgG showed a significant decrease in fluorescence signal, and therefore significantly more aggregation compared to SATA‐modified IgG. As a positive control, Bis‐NHS Ester reagent was added to crosslink IgG after conjugation to the liposome surface. This control resulted in a decrease of fluorescence of SATA chemistry to an equal level as DBCO chemistry without the Bis‐NHS Ester reagent. Comparisons made by ordinary one‐way ANOVA with Tukey's multiple comparison test (*n* = 3–9 replicates per condition).

Following the observation that the DBCO moiety itself appears to drive complement activation, we next sought to understand the mechanistic details behind this phenomenon. We hypothesized the hydrophobicity of the DBCO moiety induces IgG aggregation (**Figure** **4**a), and the IgG aggregates subsequently induce complement activation.^[^ ^26^, ^27^ ^]^ Three lines of thinking support this hypothesis. First, our initial biodistribution (Figure 2c) showed that increasing the extent of modification from 3 to 12 DBCO molecules per IgG molecule led to a 58% increase in lung uptake. Second, the in vitro studies (Figure 3a) confirmed that increased modification of IgG with DBCO results in increased complement activation. Third, the molecular structure of DBCO, which contains multiple hydrocarbon rings, suggests it is hydrophobic. We estimated hydrophobicity by calculating the partition coefficient between octanol and water (logP) using the online tool SwissADME.^[^ ^35^ ^]^ The tool predicts that DBCO possesses a logP that is 1.59 logarithmic units greater than SATA, which equates to a 39‐fold greater hydrophobicity (Figure 4b).

Previous studies have explored the effect of surface hydrophobicity on complement activation and suggested that more hydrophobic surfaces may induce greater complement activation.^[^ ^36^, ^37^, ^38^, ^39^ ^]^ However, the results are sometimes contradictory and may depend on the nanoscale structure of the surface. In the current studies, the conjugation chemistry is modifying the hydrophobicity of the IgG, rather than the surface as a whole, which may cause different complement‐related phenomena to occur.

To test our hypothesis, we measured the aggregation of IgG via HPLC‐SEC. IgG was modified with either DBCO or SATA (IgG(DBCO‐3) and IgG(SATA‐3)). Unmodified IgG and IgG aggregated via heat treatment^[^ ^40^ ^]^ were used as negative and positive controls, respectively. DBCO and SATA caused minimal aggregation, with all conditions containing less than 2% aggregates (Figure 4c,d). Although the difference between unmodified IgG and IgG(SATA‐3) is statistically significant (p = 0.002), the low percentage of aggregates in IgG(SATA‐3) are unlikely to be biologically significant.^[^ ^41^ ^]^ As discussed previously, IgG size was also measured using DLS, and the results showed minimal levels of IgG aggregation after modification with either DBCO or SATA (Figure S1, Supporting Information).

Seeing that neither DBCO nor SATA induced aggregation of solution‐phase IgG, we hypothesized that aggregation may only occur on the *surface* of the nanoparticle. The HPLC‐SEC and DLS assays only measure IgG *in solution* (i.e., not conjugated to a liposome). Surfaces are known to act as nucleation sites for aggregate formation.^[^ ^42^ ^]^ Analyzing protein aggregation on a nanoparticle surface is technically challenging, and to our knowledge no such methods exist. We adapted fluorescence quenching techniques, which have been used to study protein‐protein interactions, to assess protein aggregation on liposome surfaces. We modified IgG with either AlexaFluor‐488 (IgG‐AF488) or a quenching molecule (IgG‐TQ2). A 50/50 mixture of IgG‐AF488 and IgG‐TQ2 was then conjugated to liposomes using either DBCO or SATA chemistry. If aggregation occurs, then IgG‐AF488 and IgG‐TQ2 come into close proximity, resulting in quenching of the fluorescence signal (Figure 4e). As an internal control, we used a 50/50 mixture of IgG‐AF488 and IgG with *no quencher*. The difference in fluorescence signal between the control group (with no quencher) and the group with quencher was used to quantify aggregation.

SATA chemistry caused no change in fluorescence signal, but DBCO chemistry caused a 13% decrease in fluorescence signal compared to non‐quencher control, indicating that DBCO chemistry does induce IgG aggregation (Figure 4f). To validate the method and to establish guides for how fluorescence quenching correlates to the extent of aggregation, we tested two positive controls. First, we used thermal heat treatment to induce IgG aggregation. This control was done on IgG in solution (not conjugated to liposomes) because the thermal treatment may also disrupt the liposome structure. Heat‐induced aggregation resulted in a 27% decrease in fluorescence signal (Figure 4f). As a second positive control, we used Bis‐NHS Ester to covalently cross‐link IgG molecules after they are conjugated to the liposome surface. When Bis‐NHS Ester was used in the SATA condition, it induced a 13% decrease in fluorescence signal – nearly *identical* to DBCO chemistry without Bis‐NHS Ester crosslinker (Figure 4f). Together, the positive controls suggest that 13% decrease in fluorescence is the maximal quenching that can be achieved by IgG *on a surface*. Greater quenching is possible in solution, likely due to the formation of 3D aggregates, and therefore more numerous interactions between IgG‐AF488 and IgG‐TQ2. A review of FRET studies shows that FRET efficiencies are typically in the range of 0.1–0.4,^[^ ^43^ ^]^ which is in‐line with our results. Remarkably, DBCO chemistry alone induces the maximal 13% fluorescence quenching, indicating significant aggregation caused by the presence of the DBCO reactive moiety.

As an orthogonal method for measuring protein aggregation on the liposome surface, we utilized the aggregation‐sensitive dye 8‐anilino‐1‐naphthalenesulfonic acid (ANSA) (Figure S13, Supporting Information). The fluorescence intensity of ANSA increases in hydrophobic environments and therefore can be used to measure protein aggregation or unfolding, as these phenomena tend to expose hydrophobic pockets. ANSA was added to IgG or IgG‐liposomes, made using either DBCO‐azide or SATA‐maleimide chemistry. ANSA fluorescence increased for IgG‐liposomes compared to IgG alone. This increase was greatest for DBCO‐azide chemistry, indicating the increased accessibility of hydrophobic domains when IgG(DBCO‐12) is conjugated to liposomes and suggesting greater IgG aggregation when DBCO‐azide conjugation is used.

### 2.5 SATA/Thiol‐Maleimide Conjugation Chemistry Induces Complement Activation via Maleimide First Binding to Albumin in Plasma, with Subsequent Attack of the Immobilized Albumin by C3

**Figure 5:**
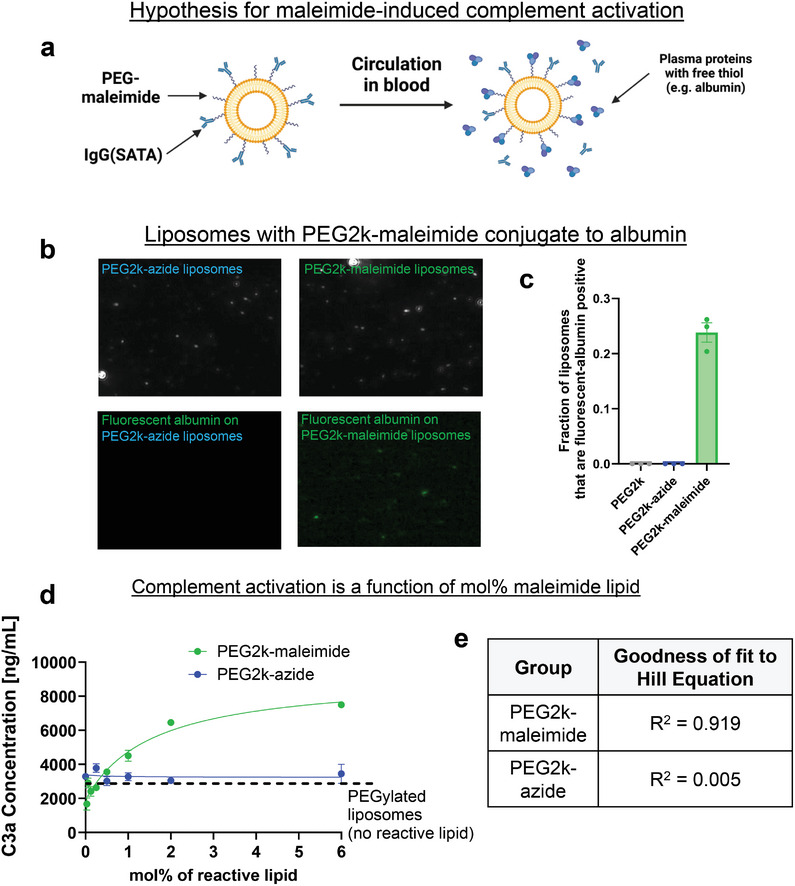
SATA/thiol‐maleimide conjugation chemistry induces complement activation via maleimide first binding to albumin in plasma, with subsequent attack of the immobilized albumin by C3. (a) Schematic depicting our hypothesis that maleimide reacts non‐specifically with thiol‐containing proteins in the blood, mainly albumin. (b) Liposomes were incubated with fluorescent mouse albumin, then analyzed via nanoparticle tracking analysis (NTA), allowing visualization of the fluorescence on individual particles. Example primary data shows total particle count (top row) and particles bound to fluorophore‐labeled albumin (bottom row). A fluorescent signal is visible in liposomes containing PEG2k‐maleimide, but not those with PEG2k‐azide. (c) Bar graph quantifying the fraction of liposomes that are fluorescent, indicates that liposomes with PEG2k‐maleimide conjugate to fluorescent albumin (n = 3 replicates per condition). (d) Liposomes containing a varying mol% of reactive lipid were incubated with serum and the concentration of C3a, a product of complement activation, was measured (*n* = 4–5 per condition). As the mol% of PEG2k‐maleimide increases, there is a corresponding rise in C3a concentration. In contrast, increasing mol% of PEG2k‐azide shows no significant change in C3a concentration. (e) The data in (d) were fit to the Hill Equation. The low R^2^ value for PEG2k‐azide indicates a poor fit and suggests complement activation is not affected by mol% of PEG2k‐azide.

Having investigated the mechanism of DBCO‐induced complement activation, we moved on to SATA/thiol‐maleimide conjugation chemistry. We hypothesized that SATA/thiol‐maleimide conjugation chemistry induces complement activation via non‐specific conjugation of proteins containing free thiols to maleimide on the liposome surface (**Figure** **5**a). Two lines of thinking also support this hypothesis. First, the in vitro studies (Figure 3b) showed that increasing SATA modification of IgG from 3‐fold to 12‐fold had minimal impact on complement activation (as measured by C_max_), and maximum complement activation was achieved at very low IgG densities (as measured by EC50). These observations suggest that complement activation is not strongly affected by the SATA moiety or IgG molecules. Second, maleimide is capable of reacting with any thiols, and there are numerous thiol‐containing species in the body (e.g., cysteine, glutathione). In fact, free thiols are present at a concentration of ∼500 uM in human plasma, which can potentially react with liposomes during circulation in the blood.^[^ ^44^ ^]^ Studies with ADCs have identified the potential of maleimide to cross‐react with cysteine‐containing molecules in vivo.^[^ ^8^ ^]^

We tested our hypothesis by investigating the reaction of liposomes with albumin. Albumin is by far the greatest source of thiols in the blood, accounting for ≈80% of free thiols in human plasma.^[^ ^44^ ^]^ We incubated liposomes with fluorophore‐labeled mouse serum albumin and then used nanoparticle tracking analysis (NTA) to visualize and count individual nanoparticles (Figure 5b). Total particle count is measured using light scattering (top row) and fluorescent albumin‐positive particles are measured using a fluorescent filter (bottom row). The fraction of liposomes positive for fluorophore‐labeled albumin can then be calculated (Figure 5c) Liposomes with maleimide reactive groups (PEG2k‐maleimide) reacted strongly to albumin, with 24% of liposomes being fluorophore positive. In contrast, liposomes with azide (PEG2k‐azide) or no reactive group (PEG2k) accumulated no fluorescent signal. The same phenomenon of albumin binding to maleimide liposomes was observed with bovine serum albumin (BSA) suggesting the generalizability of this observation to albumin from multiple species (Figure S14, Supporting Information). The same study was done using Iodine‐125 (I‐125) radiolabeled BSA in the presence of fresh mouse serum, to confirm the albumin‐liposome reaction occurs in the physiologically relevant matrix of serum. 4.15% ± 0.41% of radiolabeled albumin conjugated to liposomes containing maleimide, but only 0.07% ± 0.07% of albumin conjugated to liposomes with azide groups, confirming the conjugation of albumin to maleimide liposomes occurs in serum (Figure S14, Supporting Information).

Having established that maleimide liposomes do accumulate albumin on their surfaces, we asked if this reactivity leads to increased complement activation. We produced liposomes with varying amounts of reactive group (azide or maleimide), ranging from 0% to 6 mol%. DSPE‐PEG2k with no reactive group was added to maintain a total of 6 mol% PEGylated lipid. Complement activation induced by the liposomes was measured by incubating liposomes with mouse serum in vitro and measuring the concentration of C3a, as described in Figure 3. As the molar percentage of PEG2k‐maleimide increases, there is a corresponding rise in complement activation (Figure 5d). In contrast, increasing amounts of DSPE‐PEG2k‐azide show no significant variation in complement activation compared to PEGylated liposomes with no reactive lipid, indicating a consistent baseline activation level that is not impacted by the azide group. To further quantify this observation, we fit the data to the Hill Equation, and calculated the “Goodness of Fit” via the R^2^ parameter. The low R^2^ value of 0.005 for DSPE‐PEG2k‐azide indicates a poor fit and demonstrates complement activation is not affected by mol% of DSPE‐PEG2k‐azide (Figure 5e).

### 2.6 Rational Choice of Conjugation Chemistry Based on Mechanisms of Complement Activation

**Figure 6:**
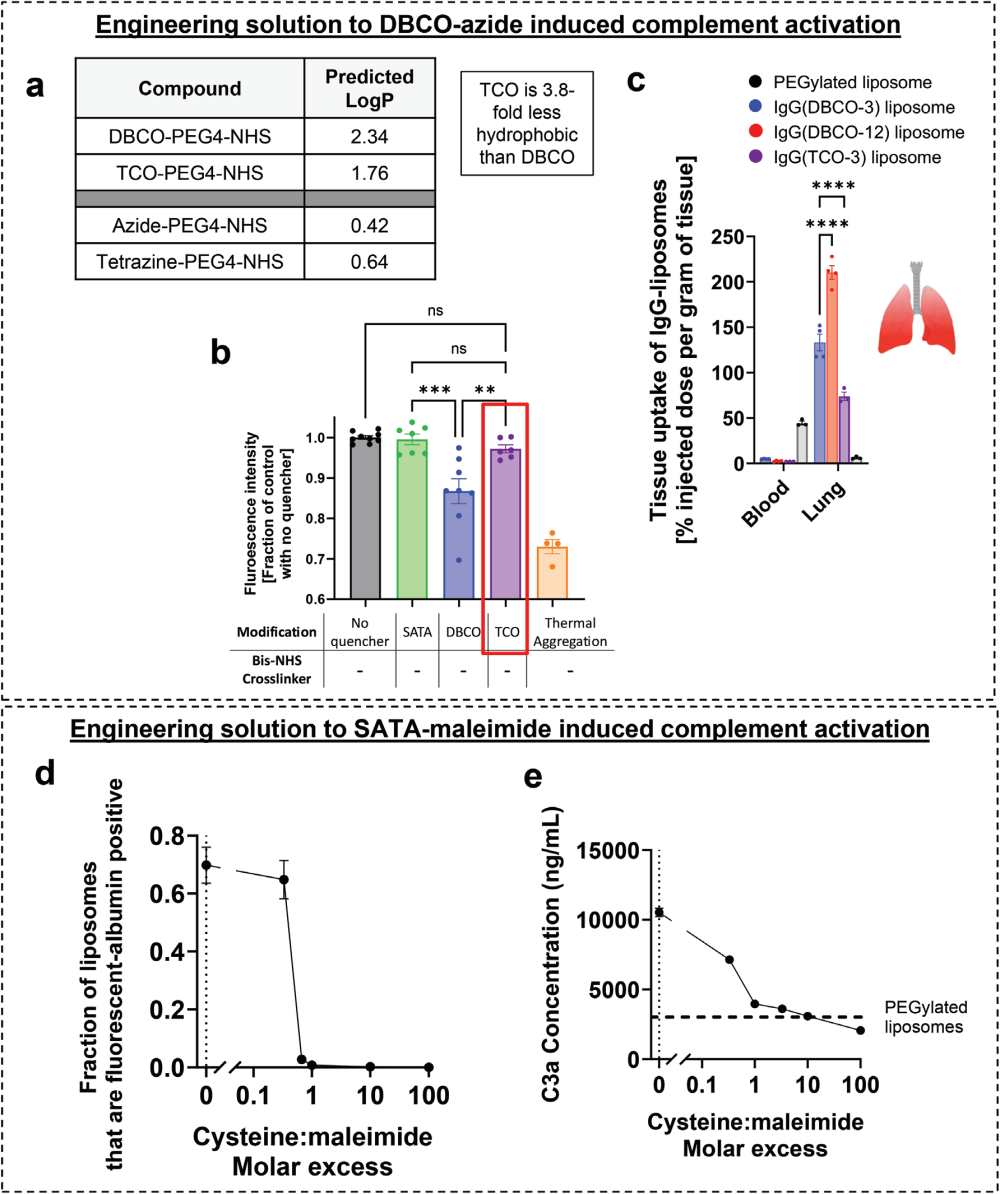
Rational choice of conjugation chemistry based on mechanisms of complement activation. Based on the mechanisms elucidated above, we devised engineering solutions to reduce complement activation for DBCO (a–c) and SATA (d,e) chemistries. (a) An alternative click chemistry, TCO‐tetrazine, utilizes reactive groups that are significantly less hydrophobic than DBCO. (b) Aggregation caused by TCO was measured using the fluorescence quenching method described in Figure 2c. TCO induced minimal quenching, which indicates minimal IgG aggregation, on par with SATA and significantly less than DBCO chemistry. Comparisons made by ordinary one‐way ANOVA with Tukey's multiple comparison test (*n* = 5–9 per condition). (c) Biodistribution of IgG‐liposomes using the same methods as Figure 1b, showing that TCO chemistry caused significantly less lung uptake compared to DBCO chemistry. Comparisons made by ordinary two‐way ANOVA with Dunnett's multiple comparison test, with IgG(DBCO‐3) liposomes as the control. Statistical significance for comparisons to PEGylated liposome control is not shown. (*n* = 3–4 mice per condition). (d) To block the reactivity of free maleimide, cysteine was added to liposomes. Cysteine addition reduced the binding of BSA to maleimide‐liposomes in a dose‐responsive manner (*n* = 3–4 per condition). (e) IgG‐liposome conjugates were reacted with varying amounts of cysteine, then incubated with serum and analyzed for C3a concentration (*n* = 4 per condition). The addition of cysteine also reduced complement activation in a dose‐responsive manner.

Armed with new mechanistic insights, we aimed to design strategies to alter antibody‐liposome conjugation chemistries to limit complement activation. To address the problem of hydrophobicity of the DBCO group, we searched for other click chemistries that utilize less hydrophobic reactive groups while still being amenable to construction of DDSs.^[^ ^21^ ^]^ We chose to investigate trans‐cyclooctene (TCO)‐tetrazine conjugation chemistry, which is a bioorthogonal click chemistry known for fast reaction kinetics. The TCO moiety contains only a single 8‐member hydrocarbon ring, indicating it will be less hydrophobic than the 3‐ring structure of DBCO (Figure S16, Supporting Information). The predicted LogP using SwissADME supports this thinking, indicating that TCO is 3.8‐fold less hydrophobic than DBCO (**Figure** **6**a).

The antibody‐liposome conjugates produced using TCO‐tetrazine chemistry were similar to those produced using DBCO‐azide and SATA‐maleimide chemistry, as measured by the size distribution, percentage of aggregated particles, and number of IgG per liposome (Figure S17, Supporting Information). We used the fluorescence quenching assay described in Figure 4e to measure the effect of TCO on IgG aggregation on liposome surfaces. TCO did not cause significant fluorescence quenching, and therefore did not induce IgG aggregation on liposomes (Figure 6b). Encouraged, we proceeded with biodistribution studies to test the in vivo effect of TCO‐tetrazine conjugation chemistry.

As with data for DBCO‐azide IgG‐liposomes in Figure 1, we IV‐injected TCO‐tetrazine IgG‐liposome conjugates into mice with acute inflammation and measured biodistribution via radioactivity. We observed that TCO‐tetrazine chemistry led to significantly less lung uptake than DBCO‐azide (74% vs 133% ID/g, Figure 6c). Although this lung uptake is much greater than C3 knock‐out conditions (≈5% ID/g, Figure 2d), indicating some level of complement opsonization and uptake by phagocytes in the lungs, it is a significant improvement compared to DBCO‐azide chemistry. Given that significant lung uptake still occurs with TCO‐tetrazine chemistry, it may be necessary to combine with other approaches, such as the use of complement regulatory proteins,^[^ ^28^, ^45^ ^]^ in order to further reduce complement activation caused by nanoparticles.

We also sought to address complement activation caused by SATA‐maleimide chemistry. We sought to eliminate the reaction of maleimide with thiols in the blood by “quenching” the maleimide with a benign thiol‐containing molecule. We chose to quench the maleimide group with cysteine, as it is an endogenous molecule that is already present in the blood. It is relatively small (MW = 121 Daltons) and therefore should not interfere with the structure of IgG or the liposome. Varying amounts of cysteine were added to liposomes containing PEG2k‐maleimide, and the liposomes were incubated with fluorescent albumin. Addition of cysteine led to a dose‐dependent inhibition of albumin binding (Figure 6d). The inflection point where albumin binding precipitously decreased was at a stoichiometric addition of cysteine to maleimide (1:1 molar ratio of cysteine to maleimide). We then tested the ability of cysteine quenching to limit complement activation. We produced IgG‐liposome conjugates using SATA/thiol‐maleimide chemistry, but after conjugation of IgG, we quenched free maleimide groups with addition of cysteine. Complement activation was measured via the in vitro assay in mouse serum described previously (Figure 3). We observed that cysteine quenching led to a dose‐dependent decrease in C3a concentration (Figure 6e). A stoichiometric addition of cysteine to maleimide decreased complement activation down to the level of PEGylated liposomes with no reactive moieties, indicating this is a practical method for limiting complement activation caused by reactive maleimide groups on a nanoparticle surface.

## 3 Conclusion

Conjugation chemistry has made tremendous progress in recent decades, enabling a wide variety of DDS's to make a clinical impact. Conjugation chemistry will similarly be important in the numerous antibody‐targeted RNA‐LNPs now in development in the boom following the COVID‐19 vaccine's success. However, we have shown that two of the most popular conjugation chemistries directly impact activation of complement by antibody‐conjugated nanoparticles, which in turn led to large increases in toxicity and changes in biodistribution. This was not previously suspected, because none of the three complement pathways (classical, alternative, or lectin) are directly activated by reactive moieties involved in bioconjugate chemistry. The mechanisms of complement activation were quite distant between the conjugation chemistries, illustrating the challenges of avoiding complement activation. Nonetheless, we engineered improvements to both conjugation chemistries that enabled large reductions in complement activation and its downstream consequences. These results suggest that further mechanistic studies and optimizations of conjugation chemistry could greatly improve the safety and efficacy of targeted nanomedicine.

## 4 Experimental Section

### Materials

DPPC (dipalmitoyl phosphatidylcholine), cholesterol, DSPE‐PEGk‐maleimide (1,2‐distearoyl‐sn‐glycero‐3‐phosphoethanolamine‐N‐[maleimide(polyethylene glycol)‐2000]), DSPE‐PEG2k‐Amine (1,2‐distearoyl‐sn‐glycero‐3‐phosphoethanolamine‐N‐[amino(polyethylene glycol)‐2000] (ammonium salt)), DSPE‐PEG2k‐azide (1,2‐distearoyl‐sn‐glycero‐3‐phosphoethanolamine‐N‐[azido(polyethylene glycol)‐2000] (ammonium salt)), and 18:1 PE‐TopFluor AF594 (1,2‐dioleoyl‐sn‐glycero‐3‐phosphoethanolamine‐N‐(TopFluor AF594) (ammonium salt)) were purchased from Avanti Polar Lipids (Alabaster, Alabama). Chloroform and bovine serum albumin (Fraction V, Cold‐ethanol precipitated, not delipidated) were purchased from Fisher Scientific (Hampton, New Hampshire).

DBCO‐PEG4‐NHS Ester (dibenzocyclooctyne‐PEG4‐N‐hydroxysuccinimidyl ester) was purchased from Conju‐Probe, LLC (San Diego, California). TCO‐PEG4‐NHS Ester and Tetrazine‐PEG5‐NHS Ester were purchased from Click Chemistry Tools (Scottsdale, Arizona). Alexa Fluor 488 (AF488)‐NHS Ester, SATA‐PEG4‐NHS Ester (N‐succinimidyl‐S‐acetyl (thio tetraethylene glycol)), Zeba Spin desalting columns (7K MWCO, 0.5 mL), and rat IgG were purchased from ThermoFisher Scientific (Waltham, Massachusetts). Murine IgG1 (Clone MOPC‐21, Catalog BE0083) and murine IgG2a (Clone C1.18.4, Catalog BE0085) were purchased from BioXCell (Lebanon, New Hampshire. Tide Quencher 2WS (TQ2)‐NHS Ester was purchased from AAT Bioquest (Pleasanton, California). Bis‐PEG3‐NHS Ester, TAMRA‐PEG4‐tetrazine, and TAMRA‐PEG2‐maleimide were purchased from BroadPharm (San Diego, California). Amicon Ultra Centrifugal filters and mouse serum albumin (not delipidated) were from Millipore‐Sigma (Burlington, Massachusetts). Sepharose 4B‐Cl resin was purchased from GE Healthcare (Pittsburg, Pennsylvania).

Fetal bovine serum was purchased from Corning (Glendale, Arizona). Collagenase, Type 1, and Fc block (Clone 93, Catalog 16‐0161‐82) were purchased from ThermoFisher Scientific. αCD45‐BUV395 (Clone 30‐F11, Catalog 564 279) was purchased from BD Biosciences (Franklin Lakes, New Jersey). αLy6G‐AlexaFluor700 (Clone 1A8, Catalog 127 622), αCD64‐PE/Cy7 (Clone: X54‐5/7.1, Catalog 139 309), and αEpCAM/CD326‐BrilliantViolet711 (Clone G8.8, Catalog 118 233) were purchased from BioLegend (San Diego, California). Lipopolysaccharide (LPS, from E. coli B4) and Cobra Venom Factor Naja naja kaouthia were purchased from Sigma–Aldrich (St. Louis, Missouri). Mouse Pie Cage for Aerosol Delivery was purchased from Braintree Scientific, Inc (Braintree MA). Aeroneb Lab Nebulizer was purchased from Kent Scientific (Torrington, Connecticut).

Streptavidin HRP Solution, TMB Substrate Reagent Set, OPtEIA Assay Diluent, rat anti‐mouse C3a (Clone I87‐1162, Catalog 558 250), rat anti‐mouse C3a Biotin (Clone I87‐419, Catalog 558 251), native mouse C3a (Catalog 558 618), rat anti‐mouse C5a (Clone I52‐1486, Catalog 558 027), rat anti‐mouse C5a biotin (Clone I52‐278, Catalog 558 028), recombinant mouse C5a (Catalog 622 597), and FUT‐175 (Futhan) (Catalog 552 035) were purchased from BD Biosciences (Franklin Lakes, New Jersey). [125I]Na and Pierce lodogen radiolabeling reagent was purchased from Perkin Elmer (Waltham, Massachusetts).

### Liposome Production and Characterization

Liposomes were produced using the thin film hydration method. Lipids were dissolved in chloroform and combined in a borosilicate glass tube. The lipid formulation for all experiments was: 54 mol% DPPC, 40 mol% cholesterol, and 6 mol% DSPE‐PEG2k with the appropriate reactive group (azide, amine, or maleimide) for each experiment. Chloroform was evaporated by blowing nitrogen over the solution until visibly dry (≈15 min) then putting the tube under vacuum for >1h. Dried lipid films were hydrated with 1X PBS, pH 7.4 to a total lipid concentration of 20 mm (0.5 to 1 mL volume after hydration). The rehydrated lipid solution was vortexed and sonicated in a bath sonicator until visually homogeneous (≈1 min each of vortexing and sonication). The solution was then extruded 21 times through a 0.2 µm polycarbonate filter. Tetrazine‐functionalized liposomes were created by adding tetrazine‐PEG5‐NHS Ester (at 15 mm in DMSO) with amine‐functionalized liposomes, at a 1:10 molar ratio (tetrazine:amine), incubating at ambient temperature for 1 h, then purifying using a Zeba Spin desalting column.

The size and polydispersity of nanoparticles were measured using Dynamic Light Scattering (DLS) and Nanoparticle Tracking Analysis (NTA). DLS measurements were performed using a Zetasizer Nano ZSP (Malvern Panalytical, Malvern, UK). NTA measurements were performed using a NanoSight NS300 (NanoSight, Salisbury, UK).

### Modification of IgG

IgG were modified using DBCO‐PEG4‐NHS Ester, SATA‐PEG4‐NHS Ester, or TCO‐PEG4‐NHS Ester according to the manufacturer's protocol. Briefly, the NHS Ester reagent (at 15 mm in DMSO) was added to IgG (at 2 to 5 mg mL^−1^) at a 5:1 or 20:1 molar ratio (NHS Ester:IgG). To track conjugation to liposomes, IgG was fluorescently modified. Alexa Fluor 488 (AF488)‐NHS Ester reagent (at 8 mm in DMSO) was added to IgG at a 0.1:1 molar ratio (NHS Ester:IgG) concurrently with modification with DBCO, SATA, or TCO. After reaction for 30 min at ambient temperature, modified IgG were purified from unreacted NHS Ester reagents using an Amicon Ultracel‐50 kDa membrane filter.

For IgG modified with SATA, the acetylated sulfhydryls in the SATA group were deprotected to sulfhydryl groups immediately before conjugation to maleimide liposomes. For deprotection, N‐hydroxylamine (at 0.5 m in PBS) was added at a 1:10 (hydroxylamine:IgG) volume ratio to the IgG solution, per manufacturer's protocol. After 2 h at ambient temperature, the solution was purified using a Zeba Spin desalting column.

For IgG modified with DBCO, the number of DBCO per IgG was measured using UV–vis spectroscopy, as described previously and explained in Figure S2 (Supporting Information).^[^ ^46^ ^]^ For IgG modified with SATA or TCO, the number of reactive groups per IgG was measured using fluorescent tracers with the appropriate reactive group. TAMRA‐maleimide or TAMRA‐tetrazine (dissolved in DMSO to 1 mg mL^−1^) were added to deprotected IgG‐SATA or IgG‐TCO, respectively, at a 30‐fold molar excess. The mixture was reacted at 4 °C for 16 h (overnight) and purified using an Amicon Ultracel‐50 kDa membrane filter. The number of TAMRA (and therefore SATA or TCO) molecules per IgG was measured using UV–vis spectroscopy, as described in Figure S2 (Supporting Information).

### Radiolabeling of IgG and Albumin

Antibodies and albumin were radioiodinated with [125I]Na using Pierce lodogen radiolabeling reagent and purified using Zeba desalting spin columns. Radiochemical purity was assessed via TLC using a mobile phase of 75% methanol:25% NH_4_ acetate, and confirmed >90% in all cases. Appropriate environmental health and safety procedures were followed for handling of radioactive material.

### Conjugation of IgG to Liposomes

IgG modified with DBCO, TCO, or SATA/thiol were conjugated to azide‐functionalized, tetrazine‐functionalized, or maleimide‐functionalized liposomes, respectively. Liposome concentration (in number of liposomes per mL) was measured using NTA and IgG concentration was measured using NanoDrop spectrophotometer (ThermoFisher, Waltham, Massachusetts). IgG was added to liposomes to achieve the desired IgG density (in the number of IgG molecules per liposome). Conjugation efficiency was initially measured using a test batch, and subsequent experiments were adjusted to account for the conjugation efficiency. For example, if IgG(DBCO‐3) is estimated to have 35% conjugation efficiency and the target coating density is 100 IgG per liposome, then an addition of 285 IgG per particle will be calculated. For DBCO‐azide and SATA/thiol‐maleimide chemistry, IgG and liposomes were incubated at 4 °C for 16 h (overnight). For TCO‐tetrazine chemistry, IgG and liposomes were incubated at 4 °C for 15 min.

After incubation, the reaction mixtures were purified by size exclusion chromatography (SEC). The IgG‐liposome conjugates were eluted through a column containing 20 mL of Sepharose 4B‐CL resin, the column was eluted with PBS, and eluent was collected into 24 1‐mL fractions. IgG conjugation was quantified by tracing ligand fluorescence or radioactivity. Efficiency of conjugation reaction was defined as the ratio of the area under the curve of the fluorescence signal in the liposome peak (fractions 6–9) over the sum of that peak combined with the free protein peak (fractions 11–16). Fractions containing IgG‐liposome conjugates were collected and re‐concentrated using Amicon filtration devices, then measured again for size and concentration using DLS and NTA.

### Biodistribution of IgG‐Liposome Conjugates

IgG‐liposome conjugates with radiolabeled IgG (125I‐IgG) were intravenously injected in naive or lipopolysaccharide (LPS) treated groups. The liposome dose was 3 mg kg^−1^ lipid, which is ≈1 × 10^11^ liposomes per 25 gram mouse. Particle dose was controlled using particle concentration measured by NTA. The radioactivity does was ≈0.1uCi per animal. For LPS groups, mice were anesthetized with 3% isoflurane, and LPS from E. coli strain B4 was administered at 2 mg kg^−1^ in 100 µL PBS via intravenous injection (retro‐orbital) 5 h prior to liposome injection. After 5 h, mice were anesthetized with 3% isoflurane and were injected intravenously (retro‐orbital) with IgG‐liposome conjugates. The animals were euthanized 30 min after nanoparticle injection. Blood was collected from the inferior vena cava (IVC) and the organs of interest were harvested, rinsed with saline, blotted dry, and weighed. The radioactivity in the blood and tissues was measured using a Wizard 2470 Automatic Gamma Counter (PerkinElmer, Waltham, Massachusetts). Radioactivity measurements and organ weights were used to calculate the tissue biodistribution in percent of injected dose per organ or percent of injected dose per gram of tissue. The total injected dose was measured prior to injections.

### Cell‐Type Distribution of IgG‐Liposome Conjugates via Flow Cytometry

Liposomes were produced using the thin film method and conjugated to IgG as described above. A fluorescent lipid was included in the formulation to facilitate tracing of the liposomes. The lipid formulation was: 53.7 mol% DPPC, 0.3 mol% 18:1 PE‐TopFluor AF594, 40 mol% cholesterol, and 6 mol% DSPE‐PEG2k with the appropriate reactive group (azide, amine, or maleimide). The liposome dose and experimental timeline were identical to the biodistribution studies described above. Upon euthanasia of mice, the lungs were perfused by inserting an 18 gauge need into the right ventricle and perfusing 5 mL of with cold PBS at 25 cm H_2_O of hydrostatic pressure. The lungs were collected, triturated, and incubated in a digestive solution of 2 mg mL^−1^ collagenase and 100 uL of 2.5 mg mL^−1^ DNase at 37 °C for 45 min, to prepare a single‐cell suspension. The cells were strained through a 70 uL cell strainer and washed with PBS. 1 mL ACK lysis buffer was added to the cells and incubated for 5 min on ice to lyse any remaining red blood cells. The cells were washed with FACS buffer (1% Fetal Bovine Serum, 1 mm EDTA, in PBS), counted using an automated cell counter (Countess, Thermo Fisher), and diluted to a concentration of 1 × 10^6^ cells mL^−1^. Cells were incubated with Fc block for 15 mins at 4 °C and washed with FACS buffer. Cells were then incubated with the following fluorophore‐conjugated antibodies for 30 min at 4 °C: αCD45‐BUV395, αLy6G‐AF488, αCD64‐PE/Cy7, αCD31‐APC, αEPCAM‐BV711. The cells were then washed with FACS buffer, fixed with 4% paraformaldehyde (PFA), and analyzed by flow cytometry (LSR Fortessa, BD BioSciences).

### Nebulized LPS Model

Mice were exposed to nebulized LPS in a “whole‐body” exposure chamber, with separate compartments for each mouse. LPS was reconstituted in PBS to 10 mg mL^−1^ and stored at −80C until use. Immediately before nebulization, LPS was thawed and diluted to 2 mg mL^−1^ with PBS. LPS was aerosolized via mesh nebulizer connected to the exposure chamber, with air flow through the chamber at 3 liters per minute. 5 mL of 2 mg mL^−1^ LPS was used to induce the injury. Nebulization was performed until all liquid was nebulized (≈20 min).

### Predicted LogP Calculation

The LogP of reactive moieties was calculated using the online tool SwissADME.^[^ ^35^ ^]^ The SMILES (Simplified Molecular Input Line Entry System) used for each chemical were as follows:

DBCO‐PEG4‐NHS: C1CC(= O)N(C1 = O)OC(= O)CCOCCOCCOCCOCCNC(= O)CCC(= O)N2CC3 = CC = CC = C3C#CC4 = CC = CC = C42

SATA‐PEG4‐NHS: O = C(ON1C(CCC1 = O) = O)CCOCCOCCOCCOCCSC(C) = O

Thiol‐PEG4‐NHS: O = C(CCOCCOCCOCCOCCS)ON1C(CCC1 = O) = O

TCO‐PEG4‐NHS: C1CC = CCCC(C1)OC(= O)NCCOCCOCCOCCOCCC(= O)ON2C(= O)CCC2 = O

Azide‐PEG4‐NHS: C1CC(= O)N(C1 = O)OC(= O)CCOCCOCCOCCOCCN = [N+] = [N‐]

Maleimide‐PEG4‐NHS: O = C1C = CC(N1CCOCCOCCOCCOCC(ON2C(CCC2 = O) = O) = O) = O

Tetrazine‐PEG4‐NHS: O = C(ON1C(CCC1 = O) = O)CCOCCOCCOCCOCCC(NCC2 = CC = C(C3 = NN = CN = N3)C = C2) = O

### HPLC‐SEC

IgG aggregation was measured via HPLC‐SEC. Liquid chromatography was performed on an Agilent 1260 Infinity II LC system, with a quaternary pump, autosampler, and diode array detector (DAD), using an AdvanceBio SEC Column, 300Å, 4.6 × 300 mm, 2.7 µm (Agilent). Mobile phase was 0.1 m sodium phosphate buffer, pH 6.8, pumped at 0.35 mL min^−1^. IgG was detected at a wavelength of 280 nm. IgG eluted at ≈6.5 min. Percent of IgG that is aggregated was calculated as the Area Under Curve (AUC) of any aggregate peaks divided by the total AUC for all peaks. As a positive control, IgG aggregation was induced by heating IgG at 65 °C for 20 min.^[^ ^40^ ^]^

### Surface Aggregation Fluorescence Quenching Assay

For surface aggregation assays, IgG was modified with Alexa Fluor 488(AF488)‐NHS Ester or Tide Quencher 2WS(TQ2)‐NHS Ester at a 5:1 molar ratio (NHS Ester:IgG). IgG was modified with fluorescent labels at the same time as modification with DBCO, SATA, or TCO. After reaction for 30 min at ambient temperature, modified IgG were purified from unreacted NHS Ester reagents using an Amicon Ultracel‐50 kDa membrane filter.

Liposomes were made as described in “Liposome Production”, and conjugated with a total of 100 IgG molecules per liposome. Half of the IgG (50 IgG per liposome) were labeled with AF488 and the appropriate conjugation moiety (DBCO, SATA, TCO). The other half of the IgG (50 IgG per liposome) were labeled with TQ2 and the appropriate conjugation moiety. To quantify the amount of fluorescence quenching, a control group was done with half of the IgG NOT labeled with TQ2, and only labeled with the conjugation moiety. IgG‐liposome conjugation was performed according to the section above titled “Conjugation of IgG to Liposomes”. After conjugation and elution through the Sepharose SEC, the fluorescence intensity of the liposome peak was measured. To determine the extent of fluorescence quenching, the fluorescence intensity of liposomes containing TQ2 was compared to liposomes without TQ2. The fraction of fluorescence intensity compared to the control group with no quencher was reported.

To validate the assay, a positive control was included, where a 200 molar excess of Bis‐PEG3‐NHS was introduced to crosslink the IgGs after conjugation to the liposome surface, followed by a 1 h incubation in darkness at ambient temperature. Subsequently, liposomes were purified via SEC for a second time to separate free Bis‐NHS from the liposomes, effectively terminating the reaction. Fluorescence intensity of groups with and without quencher were measured and used to calculate the extent of fluorescence quenching.

As an additional positive control, the fluorescence quenching of IgG in solution was determined. A 50:50 mixture (mass:mass) of IgG labeled with AF488 and IgG labeled with TQ2 were combined in a polypropylene tube. IgG was aggregated by heating at 65 °C for 20 min.^[^ ^40^ ^]^ The fluorescence intensity of the resulting solution was measured. The extent of quenching was calculated by comparing fluorescence intensity to a control group that contained a 50:50 mixture (mass:mass) of IgG labeled with AF488 and IgG NOT labeled with TQ2.

### 8‐Anilino‐1‐Naphthalenesulfonic Acid Nanoparticle Staining

6 uL of 8‐Anilino‐1‐naphthalenesulfonic acid (ANSA) at 1 mg mL^−1^ was added to 94 uL of IgG or IgG‐liposome conjugates at a protein concentration of 1 mg mL^−1^. The mixture was incubated at room temperature for 30 min. Unreacted ANSA was removed by three centrifugations through a 100 kDa MWCO centrifugal filter (Amicon). The sample was resuspended to the original volume and fluorescence intensity was measured at an excitation of 375 nm and emission scan from 400 to 500 nm.

### Albumin Accumulation on Liposomes

Liposomes were made as described in “Nanoparticle Production”. Either mouse albumin or bovine serum albumin (neither delipidated) were fluorescently labeled with a 2:1 molar ratio of fluorophore to protein (AF488‐NHS Ester: albumin). The mixture was reacted at ambient temperature for 30 min, then purified via an Amicon 10 kDa MWCO centrifugal filter, resulting in ≈1 molecule of AF488 per molecule of albumin. 10 uL of liposomes (at 2 × 10^13^ particles per mL) were added to 90 uL of fluorescent‐albumin (at 0.75 mg mL^−1^). The liposome‐albumin mixture was incubated at 37 °C for 30 min. Unbound albumin was removed via an Amicon 100 kDa MWCO centrifugal filter. The purified liposome sample was analyzed via NTA. Nanoparticle concentration was measured both using light scatter (to measure total liposome concentration), and using a 488 nm laser / 500 nm filter (to measure the concentration of liposomes bound with fluorescent‐albumin). To quench maleimide groups with cysteine, a solution of cysteine‐HCl was prepared in PBS at 1.0 mg mL^−1^ and added to liposomes to achieve the desired molar ratio of cysteine to maleimide. After addition of cysteine, the mixture was incubated at 4 °C for 30 min, then unreacted cysteine was removed via an Amicon 100 kDa MWCO centrifugal filter.

For radiotracing studies, I‐125 labeled albumin was prepared at a protein concentration of 25 mg mL^−1^ and activity of 2uCi mL^−1^. 40 uL of I‐125 albumin was added to 160 uL of fresh mouse serum, 5 uL of liposomes, at a concentration of 2 × 10^13^ liposomes mL^−1^, were added to 95 uL of the serum/albumin mixture. The mixture was incubated at 37 °C for 30 min, and then eluted through a column containing 20 mL of Sepharose 4B‐CL resin. 1 mL fractions were collected and radioactivity was measured using a Wizard 2470 Automatic Gamma Counter.

### C3a and C5a ELISA

C3a and C5a levels in vitro and in vivo were measured via a sandwich ELISA, per manufacturer recommended assay procedures (BD Biosciences). Briefly, for in vitro measurement, serum was collected from mouse inferior vena cava with sterile syringes, and then centrifuged for 10 min at 2000 g after allowing to clot for 30 min at ambient temperature. 20 µL of fresh serum was incubated with 20 µL of IgG‐liposome conjugates (2 × 10^12^ liposomes per mL) for 15 min at 37 °C, EDTA was added to a final concentration of 20 × 10^−3^ m, to inhibit further complement activation. To inhibit the classical and lectin pathways of complement and isolate the alternative pathway, 10 uL Gelatin Veronal Buffer (GVB) with MgEGTA was added to 20 uL of serum, then 10 uL of IgG‐liposome conjugates (4 × 10^12^ liposomes per mL) were added and incubated for 15 min at 37 °C. The percentage of complement activation due to the alternative pathway was calculated as:

(1) $$\%\;\;\mathrm{Alternative}\;\;\mathrm{Pathway}=\frac{C3a\;\mathrm{concentration}\;\mathrm{with}\;\mathrm{GVB}\;\mathrm{present}}{C3a\;\mathrm{concentration}\;\mathrm{without}\;\mathrm{GVB}\;\mathrm{present}}$$

For in vivo measurement, plasma was collected from mouse inferior vena cava with EDTA coated syringes, and then chelated with 20 × 10^−3^ m EDTA and the pan‐complement inhibitor Futhan (0.05 mg mL^−1^, BD Pharmingen) to inhibit further complement activation. Plasma was separated from blood via centrifugation at 2000 g for 10 min.

### Curve Fitting

Data were fit using GraphPad PRISM 10. Data was fit to the Hill equation, with the x‐variable set to the mol% of reactive lipid (Figure 5d) or the number of IgG molecules per liposome (Figure 3c) and the y‐variable set to C3a concentration. The Hill Slope was fixed to 1.0, and the equation was fit using least squares regression, solving for three parameters: basal response (bottom), maximum response (top, or C_max_) and EC50 (x‐value that produces half‐maximum response). For Figure 4a,b, the basal response (bottom) was constrained to be equal for both data sets in the figure (i.e., IgG(SATA‐3) and IgG(SATA‐12) were constrained to produce the same basal C3a concentration when there are zero IgG per liposome)

### Ethics Statement

The mice were maintained at 22–26 °C and adhered to a 12/12 h dark/light cycle with food and water ad libitum. All animal studies were carried out in accordance with the Guide for the Care and Use of Laboratory Animals (National Institutes of Health, Bethesda, MD), and all animal protocols were approved by the University of Pennsylvania Institutional Animal Care and Use Committee. All animal experiments were carried out using male, 6–8 week old C57BL/6 mice (20–25 g), and genetically modified mice for C3KO, B6;129S4‐C3^1Crr^/J of the same age and size. (The Jackson Laboratory, Bar Harbor, ME).

### Statistics

All results are expressed as mean ± Standard Error of the Mean (SEM) unless specified otherwise. All data processing steps, sample sizes, and statistical methods are described in the relevant Experimental sections. Statistical analyses were performed using GraphPad Prism 10.2.3 (GraphPad Software) ^*^ denotes *p *< 0.05, ^**^ denotes *p* < 0.01, ^***^ denotes *p* < 0.001, ^****^ denotes *p* < 0.0001.

## Conflict of Interest

The authors declare no conflict of interest.

## Supporting information



Supporting Information

Supplemental Video 1

## Data Availability

The data that support the findings of this study are available from the corresponding author upon reasonable request.

## References

[1] R. E. Bird , S. A. Lemmel , X. Yu , Q. A. Zhou , Bioconjug. Chem. 2021, 32, 2457. 34846126 10.1021/acs.bioconjchem.1c00461

[2] A. Eras , D. Castillo , M. Suárez , N. S. Vispo , F. Albericio , H. Rodriguez , Front Chem 2022, 10, 889083. 35720996 10.3389/fchem.2022.889083 PMC9204480

[3] H. C. Kolb , M. G. Finn , K. B. Sharpless , Angew. Chem. Int. Ed. Engl. 2001, 40, 2004. 11433435 10.1002/1521-3773(20010601)40:11<2004::AID-ANIE2004>3.0.CO;2-5

[4] N. J. Agard , J. A. Prescher , C. R. Bertozzi , J. Am. Chem. Soc. 2004, 126, 15046. 15547999 10.1021/ja044996f

[5] P. Thirumurugan , D. Matosiuk , K. Jozwiak , Chem. Rev. 2013, 113, 4905. 23531040 10.1021/cr200409f

[6] M. Acchione , H. Kwon , C. M. Jochheim , W. M. Atkins , mAbs 2012, 4, 362. 22531451 10.4161/mabs.19449 PMC3355488

[7] P. Strop , S.‐H. Liu , M. Dorywalska , K. Delaria , R. G. Dushin , T.‐T. Tran , W.‐H. Ho , S. Farias , M. G. Casas , Y. Abdiche , D. Zhou , R. Chandrasekaran , C. Samain , C. Loo , A. Rossi , M. Rickert , S. Krimm , T. Wong , S. M. Chin , J. Yu , J. Dilley , J. Chaparro‐Riggers , G. F. Filzen , C. J. O'Donnell , F. Wang , J. S. Myers , J. Pons , D. L. Shelton , A. Rajpal , Chem. Biol. 2013, 20, 161. 23438745 10.1016/j.chembiol.2013.01.010

[8] B.‐Q. Shen , K. Xu , L. Liu , H. Raab , S. Bhakta , M. Kenrick , K. L. Parsons‐Reponte , J. Tien , S.‐F. Yu , E. Mai , D. Li , J. Tibbitts , J. Baudys , O. M. Saad , S. J. Scales , P. J. McDonald , P. E. Hass , C. Eigenbrot , T. Nguyen , W. A. Solis , R. N. Fuji , K. M. Flagella , D. Patel , S. D. Spencer , L. A. Khawli , A. Ebens , W. L. Wong , R. Vandlen , S. Kaur , M. X. Sliwkowski , et al., Nat. Biotechnol. 2012, 30, 184. 22267010 10.1038/nbt.2108

[9] N. Joubert , A. Beck , C. Dumontet , C. Denevault‐Sabourin , Pharmaceuticals 2020, 13, 245. 32937862 10.3390/ph13090245 PMC7558467

[10] V. Kostova , P. Désos , J.‐B. Starck , A. Kotschy , Pharmaceuticals 2021, 14, 442. 34067144 10.3390/ph14050442 PMC8152005

[11] A. C. Anselmo , S. Mitragotri , Bioeng. Transl. Med. 2021, 6, e10246. 34514159 10.1002/btm2.10246 PMC8420572

[12] K. Werengowska‐Ciećwierz , M. Wiśniewski , A. P. Terzyk , S. Furmaniak , Adv. Condens. Matter Phys. 2015, 2015, 442. 10.1088/0953-8984/28/1/015002 26569632

[13] L. Nobs , F. Buchegger , R. Gurny , E. Allémann , J. Pharm. Sci. 2004, 93, 1980. 15236448 10.1002/jps.20098

[14] J. P. Oliveira , A. R. Prado , W. J. Keijok , P. W. P. Antunes , E. R. Yapuchura , M. C. C. Guimarães , Sci. Rep. 2019, 9, 13859. 31554912 10.1038/s41598-019-50424-5 PMC6761283

[15] M. H. Jazayeri , H. Amani , A. A. Pourfatollah , H. Pazoki‐Toroudi , B. Sedighimoghaddam , Sens. Bio‐Sens. Res. 2016, 9, 17.

[16] D. Ricklin , E. S. Reis , D. C. Mastellos , P. Gros , J. D. Lambris , Immunol. Rev. 2016, 274, 33. 27782325 10.1111/imr.12500 PMC5427221

[17] Z. Wang , J. S. Brenner , AAPS J. 2021, 23, 105. 34505951 10.1208/s12248-021-00630-9 PMC8432284

[18] F. Barbero , L. Russo , M. Vitali , J. Piella , I. Salvo , M. L. Borrajo , M. Busquets‐Fité , R. Grandori , N. G. Bastús , E. Casals , V. Puntes , Semin. Immunol. 2017, 34, 52. 29066063 10.1016/j.smim.2017.10.001

[19] S. M. Moghimi , A. J. Andersen , D. Ahmadvand , P. P. Wibroe , T. L. Andresen , A. C. Hunter , Adv. Drug Delivery Rev. 2011, 63, 1000. 10.1016/j.addr.2011.06.002 21689701

[20] R. Dudchak , M. Podolak , S. Holota , O. Szewczyk‐Roszczenko , P. Roszczenko , A. Bielawska , R. Lesyk , K. Bielawski , Bioorg. Chem. 2024, 143, 106982. 37995642 10.1016/j.bioorg.2023.106982

[21] S. M. Kondengadan , S. Bansal , C. Yang , D. Liu , Z. Fultz , B. Wang , Acta Pharm. Sin. B 2023, 13, 1990. 37250163 10.1016/j.apsb.2022.10.015 PMC10213991

[22] S. J. Walsh , J. D. Bargh , F. M. Dannheim , A. R. Hanby , H. Seki , A. J. Counsell , X. Ou , E. Fowler , N. Ashman , Y. Takada , A. Isidro‐Llobet , J. S. Parker , J. S. Carroll , D. R. Spring , Chem. Soc. Rev. 2021, 50, 1305. 33290462 10.1039/d0cs00310g

[23] D. B. Kirpotin , D. C. Drummond , Y. Shao , M. R. Shalaby , K. Hong , U. B. Nielsen , J. D. Marks , C. C. Benz , J. W. Park , Cancer Res. 2006, 66, 6732. 16818648 10.1158/0008-5472.CAN-05-4199

[24] S.‐S. Kim , J. B. Harford , M. Moghe , A. Rait , E. H. Chang , Oncoimmunology 2018, 7, e1484982. 30288347 10.1080/2162402X.2018.1484982 PMC6169574

[25] W. Kamoun , E. Swindell , C. Pien , L. Luus , J. Cain , M. Pham , I. Kandela , Z. R. Huang , S. K. Tipparaju , A. Koshkaryev , V. Askoxylakis , D. B. Kirpotin , T. Bloom , M. Mino‐Kenudson , J. D. Marks , A. Zalutskaya , W. Bshara , C. Morrison , D. C. Drummond , Pharmaceutics 2020, 12, 996. 33092175 10.3390/pharmaceutics12100996 PMC7589819

[26] C. C. Squaiella‐Baptistão , J. R. Marcelino , L. E. Ribeiro da Cunha , J. M. Gutiérrez , D. V. Tambourgi , Am. J. Trop. Med. Hyg. 2014, 90, 574. 24445201 10.4269/ajtmh.13-0591 PMC3945706

[27] M. L. E. Lundahl , S. Fogli , P. E. Colavita , E. M. Scanlan , RSC Chem Biol 2021, 2, 1004. 34458822 10.1039/d1cb00067e PMC8341748

[28] Z. Wang , E. D. Hood , J. Nong , J. Ding , O. A. Marcos‐Contreras , P. M. Glassman , K. M. Rubey , M. Zaleski , C. L. Espy , D. Gullipali , T. Miwa , V. R. Muzykantov , W.‐C. Song , J. W. Myerson , J. S. Brenner , Adv. Mater. 2022, 34, 2107070. 10.1002/adma.202107070 PMC9062787 34910334

[29] J. W. Myerson , P. N. Patel , K. M. Rubey , M. E. Zamora , M. H. Zaleski , N. Habibi , L. R. Walsh , Y.‐W. Lee , D. C. Luther , L. T. Ferguson , O. A. Marcos‐Contreras , P. M. Glassman , L. L. Mazaleuskaya , I. Johnston , E. D. Hood , T. Shuvaeva , J. Wu , H.‐Y. Zhang , J. V. Gregory , R. Y. Kiseleva , J. Nong , T. Grosser , C. F. Greineder , S. Mitragotri , G. S. Worthen , V. M. Rotello , J. Lahann , V. R. Muzykantov , J. S. Brenner , Nat. Nanotechnol. 2022, 17, 86. 34795440 10.1038/s41565-021-00997-y PMC8776575

[30] A. M. Collins , Immunol. Cell Biol. 2016, 94, 949. 27502143 10.1038/icb.2016.65

[31] J. Köhl , Mol. Immunol. 2001, 38, 175. 11532279 10.1016/s0161-5890(01)00041-4

[32] L. Dézsi , T. Mészáros , E. Őrfi , T. G. Fülöp , M. Hennies , L. Rosivall , P. Hamar , J. Szebeni , G. Szénási , Molecules 2019, 24, 3283. 31505853 10.3390/molecules24183283 PMC6767111

[33] Z. Patkó , J. Szebeni , Eur. J. Nanomed. 2015, 7, 233.

[34] E. Őrfi , T. Mészáros , M. Hennies , T. Fülöp , L. Dézsi , A. Nardocci , L. Rosivall , P. Hamar , B. W. Neun , M. A. Dobrovolskaia , J. Szebeni , G. Szénási , Int. J. Nanomed. 2019, 14, 1563. 10.2147/IJN.S187139 PMC6396670 30880965

[35] A. Daina , O. Michielin , V. Zoete , Sci. Rep. 2017, 7, 42717. 28256516 10.1038/srep42717 PMC5335600

[36] M. Hulander , A. Lundgren , M. Berglin , M. Ohrlander , J. Lausmaa , H. Elwing , Int. J. Nanomed. 2011, 6, 2653. 10.2147/IJN.S24578 PMC3218579 22114496

[37] Y. S. Lin , V. Hlady , J. Janatova , Biomaterials 1992, 13, 905. 1321678 10.1016/0142-9612(92)90100-3

[38] K. N. Ekdahl , B. Nilsson , C. G. Gölander , H. Elwing , B. Lassen , U. R. Nilsson , J. Colloid Interface Sci. 1993, 158, 121.

[39] M. Toda , H. Iwata , ACS Appl. Mater. Interfaces 2010, 2, 1107. 20380387 10.1021/am900891h

[40] K. K. Ostreiko , I. A. Tumanova , Sykulev Y.uK. , Immunol. Lett. 1987, 15, 311. 3692537 10.1016/0165-2478(87)90134-9

[41] W. Wang , S. K. Singh , N. Li , M. R. Toler , K. R. King , S. Nema , Int. J. Pharm. 2012, 431, 1. 22546296 10.1016/j.ijpharm.2012.04.040

[42] M. R. G. Kopp , F. Grigolato , D. Zürcher , T. K. Das , D. Chou , K. Wuchner , P. Arosio , J. Pharm. Sci. 2023, 112, 377. 36223809 10.1016/j.xphs.2022.10.009

[43] T. Lin , B. L. Scott , A. D. Hoppe , S. Chakravarty , Protein Sci. 2018, 27, 1850. 30052312 10.1002/pro.3482 PMC6199148

[44] L. Turell , R. Radi , B. Alvarez , Biol. Med. 2013, 65, 244. 10.1016/j.freeradbiomed.2013.05.050 PMC3909715 23747983

[45] Y. Li , S. Jacques , H. Gaikwad , G. Wang , N. K. Banda , V. M. Holers , R. I. Scheinman , S. Tomlinson , S. M. Moghimi , D. Simberg , Nat. Nanotechnol. 2023, 19, 246. 37798566 10.1038/s41565-023-01514-z PMC11034866

[46] J. Wiener , D. Kokotek , S. Rosowski , H. Lickert , M. Meier , Sci. Rep. 2020, 10, 1457. 31996713 10.1038/s41598-020-58238-6 PMC6989672

