# Supplementary material for: Conjugation Chemistry Markedly Impacts Toxicity and Biodistribution of Targeted Nanoparticles, Mediated by Complement Activation
Source: Adv Mater. 2024 Dec 11;37(5):2409945. doi: 10.1002/adma.202409945 (PMC11795710; doi:10.1002/adma.202409945)
Supplement: Supplementary file 1 — Supporting Information [file ADMA-37-2409945-s002.docx]

# Supporting Information

# **Conjugation chemistry markedly impacts toxicity and biodistribution of targeted nanoparticles, mediated by complement activation**

*Michael H. Zaleski* ^a^, Liam S. Chase* ^a^, Elizabeth D. Hood^a^, Zhicheng Wang^a^, Jia Nong^a^, Carolann L. Espy^a^, Marco E. Zamora^a^, Jichuan Wu^a^, Lianne J. Morrell^a^, Vladimir R. Muzykantov^a^, Jacob W. Myerson** ^a^, Jacob S. Brenner** ^a,b^*

* These authors contributed equally

** To whom correspondence should be addressed

^a^Department of Systems Pharmacology and Translational Therapeutics, The Perelman School of Medicine, University of Pennsylvania, Philadelphia, PA, USA

^b^Department of Medicine, University of Pennsylvania, Philadelphia, PA, USA


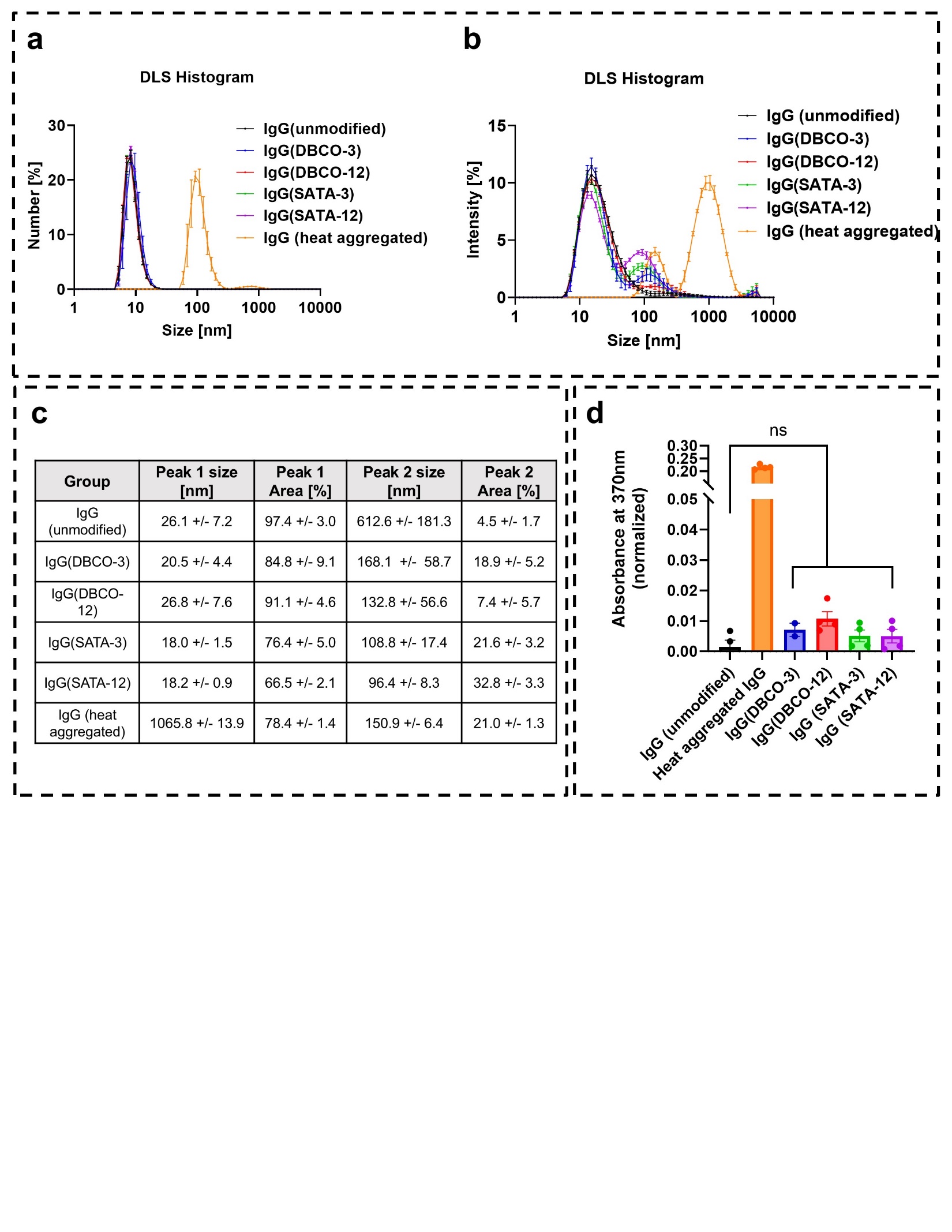


**Supplemental Figure S1. Size characterization of modified IgG.**

IgG was modified with DBCO or SATA via NHS-Ester addition, and the protein size was measured via DLS to assess the presence of protein aggregates. Heat-aggregated IgG is included as a positive control. (a) Number-based size-distribution for all conditions. IgG is approximately 10 nm in size, with no change due to modification with DBCO or SATA. (b) Intensity-based size-distribution for all conditions indicates the presence of some protein aggregates with sizes approximately 100 nm. However, we note that intensity-based size distribution data exaggerates the presence of large-sized particles, because scattering intensity scales as size to the 6th power. (c) The size and intensity percent of the two most intense peaks, from the DLS profiles in panel (b). (d) The absorbance of IgG at 370 nm was used as a measure of turbidity to assess potential IgG precipitation caused by modification. Neither modification with DBCO nor SATA caused an increase in absorbance, suggesting they do not cause IgG precipitation.


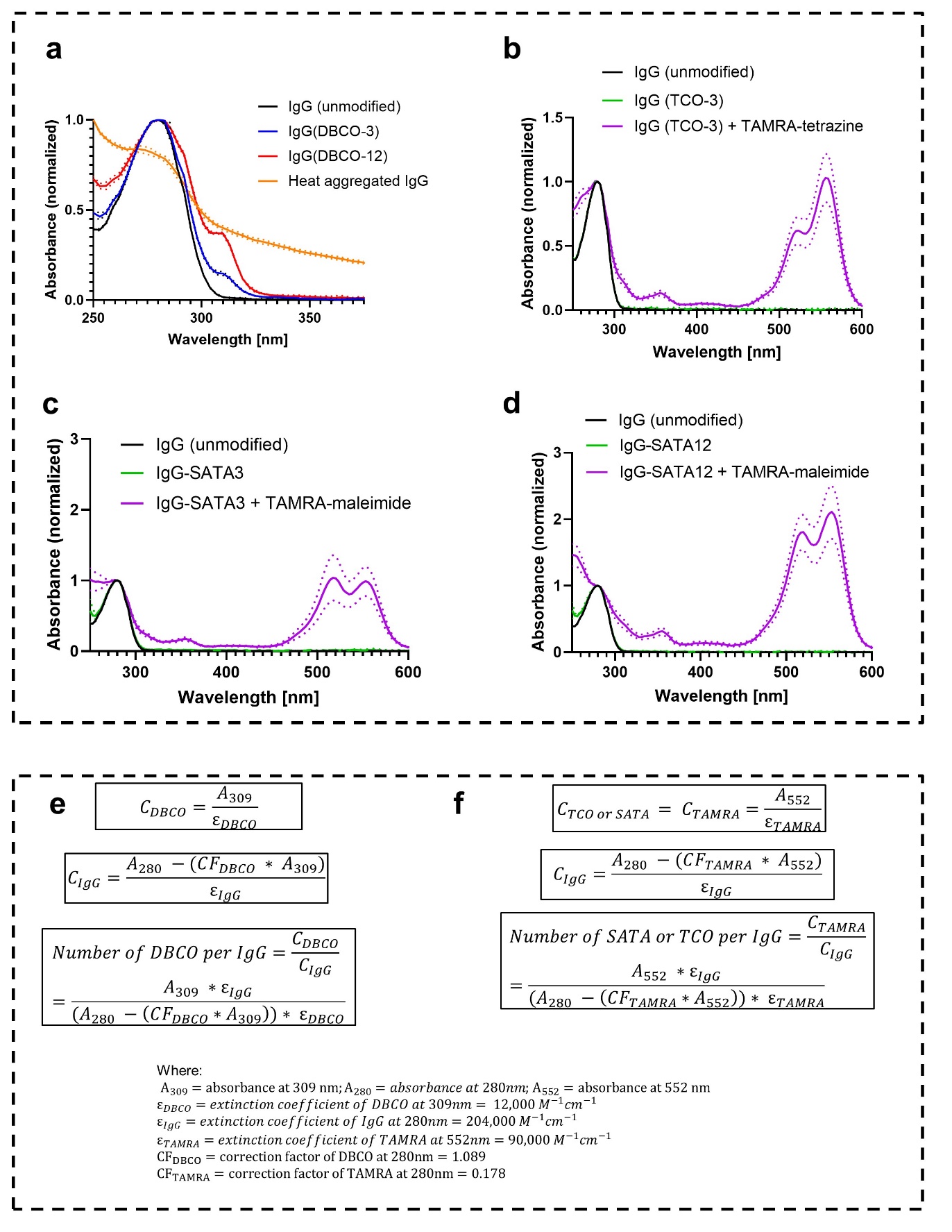


**Supplemental Figure S2. Quantification of the number of reactive groups per IgG.**

The number of DBCO per IgG was quantified using UV-Vis spectroscopy, as described in Wiener et al.[^1^](https://paperpile.com/c/6y1mkK/fwtcP) The number of SATA and TCO per IgG was quantified using a similar approach as with DBCO, but using a reactive fluorescent group as a tracer, as SATA and TCO have no intrinsic absorbance at wavelengths above 280 nm. (a) UV-Vis absorbance profile of IgG before and after modification with DBCO. Note the shoulder at 309 nm for DBCO-modified IgG, which is due to the presence of DBCO. (b) UV-Vis absorbance profile of IgG before and after modification with TCO and after reaction with TAMRA-tetrazine. (c and d) UV-Vis absorbance profile of IgG before and after modification with SATA and after reaction with TAMRA-maleimide. (e) Equations used for calculation of the number of DBCO molecules per IgG. Absorbance at 309 nm is used to calculate the DBCO concentration, using the known molar extinction coefficient of DBCO. Absorbance at 280 nm is corrected based on the DBCO concentration. Then, the corrected absorbance at 280 nm is used to calculate the concentration of IgG, using the known molar extinction coefficient of IgG. (f) Equations used for calculation of the number of TCO or SATA molecules per IgG. Absorbance at 552 nm is used to calculate the TAMRA concentration. Absorbance at 280 nm is corrected based on TAMRA concentration, and then used to calculate IgG concentration.


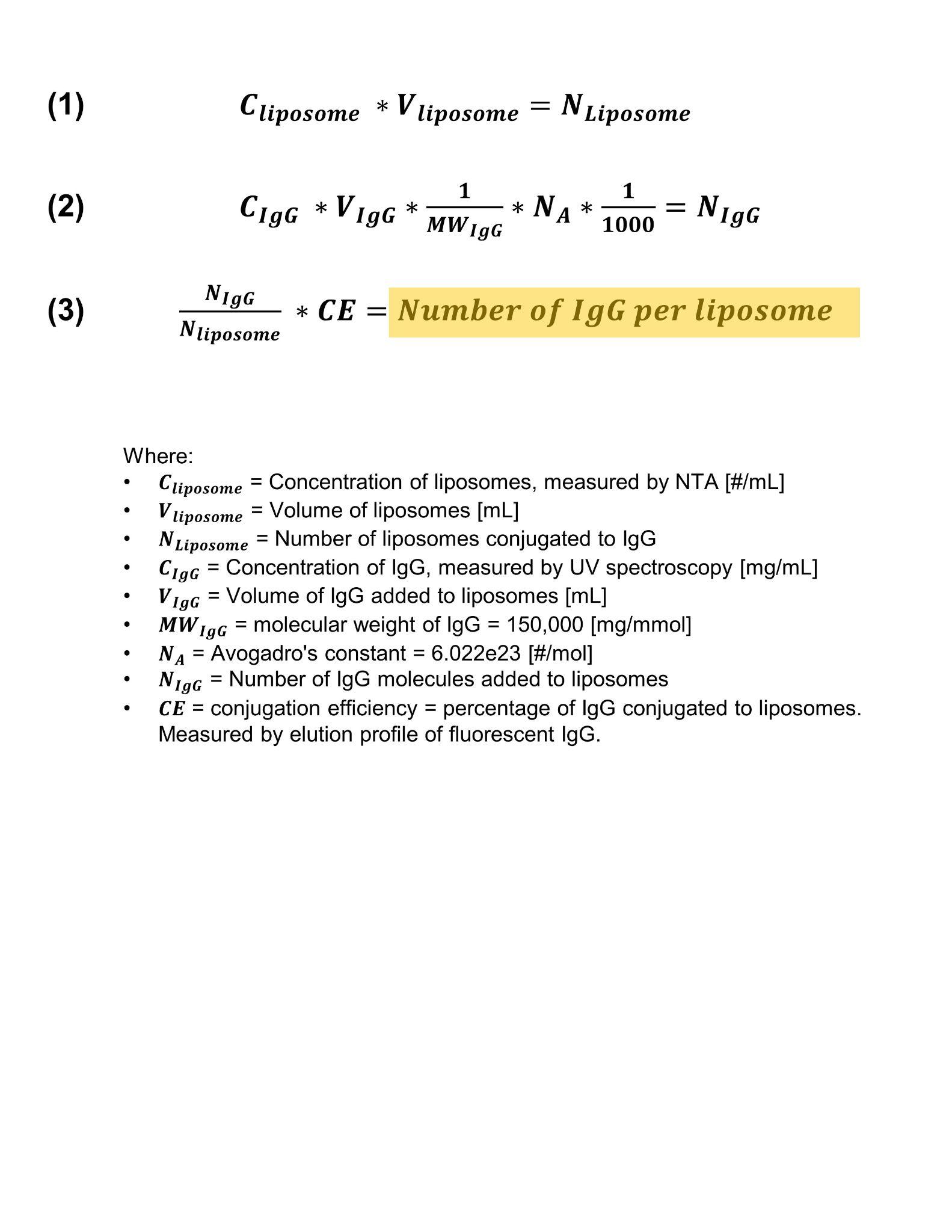


**Supplemental Figure S3. Equations for calculating the number of IgG molecules conjugated per liposome.**

The number of IgG conjugated per liposome was calculated based on known liposome concentration from NTA, known IgG concentration from Uv-Vis spectroscopy, and conjugation efficiency determined from Size-Exclusion Chromatography (SEC). (Equation 1) The number of liposomes included in the reaction was calculated from particle concentration measured by NTA and volume of liposomes used. (Equation 2) The number of IgG molecules included in the reaction was calculated from the concentration determined by UV-Vis spectroscopy and volume of IgG used. (Equation 3) The final number of IgG per liposome was calculated based on the conjugation efficiency determined from the SEC elution profile. See Figure 1b for an example elution profile. Based on prior experience with both alkyne-azide and thiol-maleimide conjugation reactions, the approximate conjugation efficiency was known (See Supplemental Figure S6a). Therefore, reactions could be planned so that the final yield would be approximately 200 antibodies conjugated per liposome for each condition.


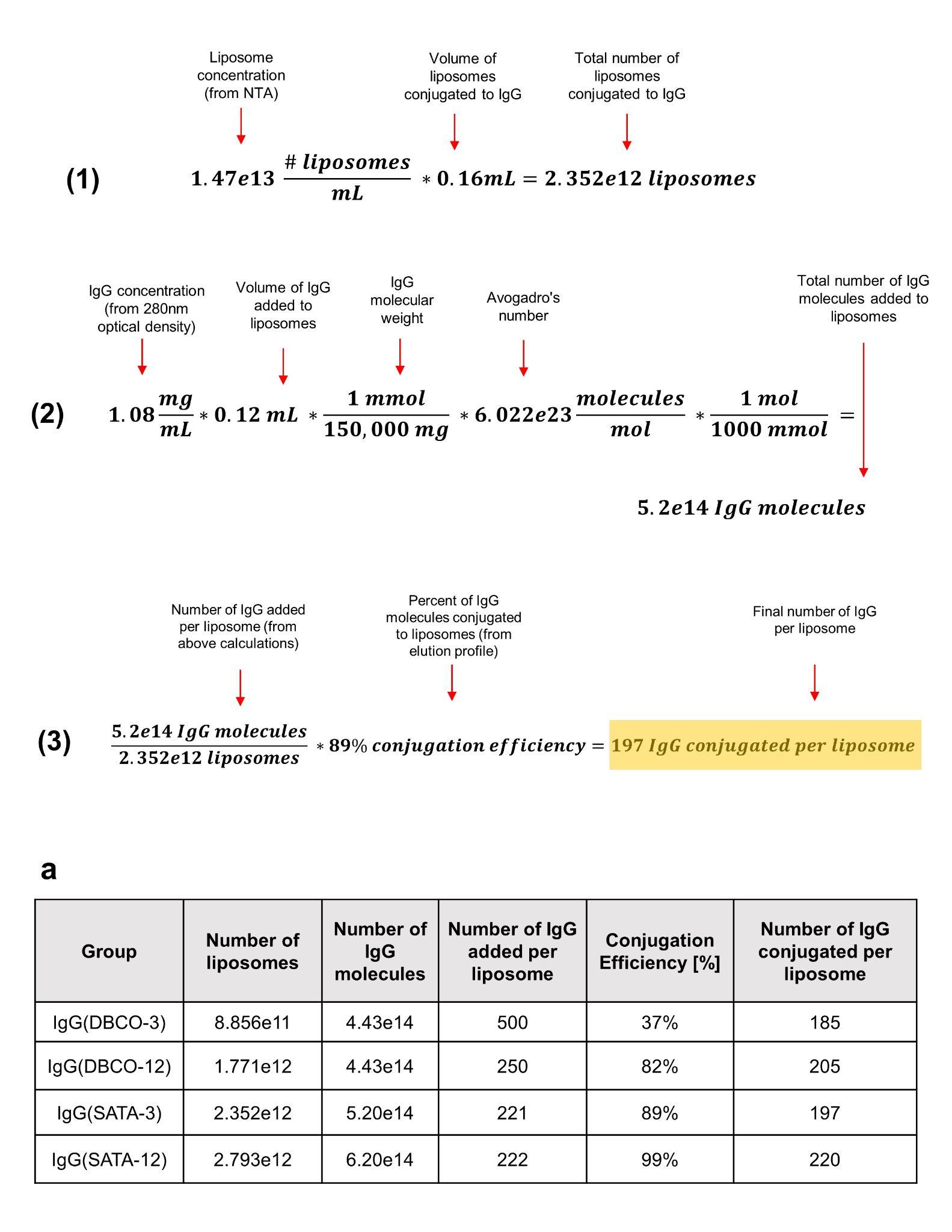


**Supplemental Figure S4. Example calculation of the number of IgG molecules per liposome.**

An example calculation for the number of IgG molecules per liposome is shown, using the equations provided in Supplemental Figure 3. (a) Table of representative calculations for each conjugation chemistry. Note that the conjugation efficiency is different for each chemistry, and therefore the total number of IgG added per liposome is adjusted to account for this difference and still yield a final density of 200 IgG molecules per liposome.


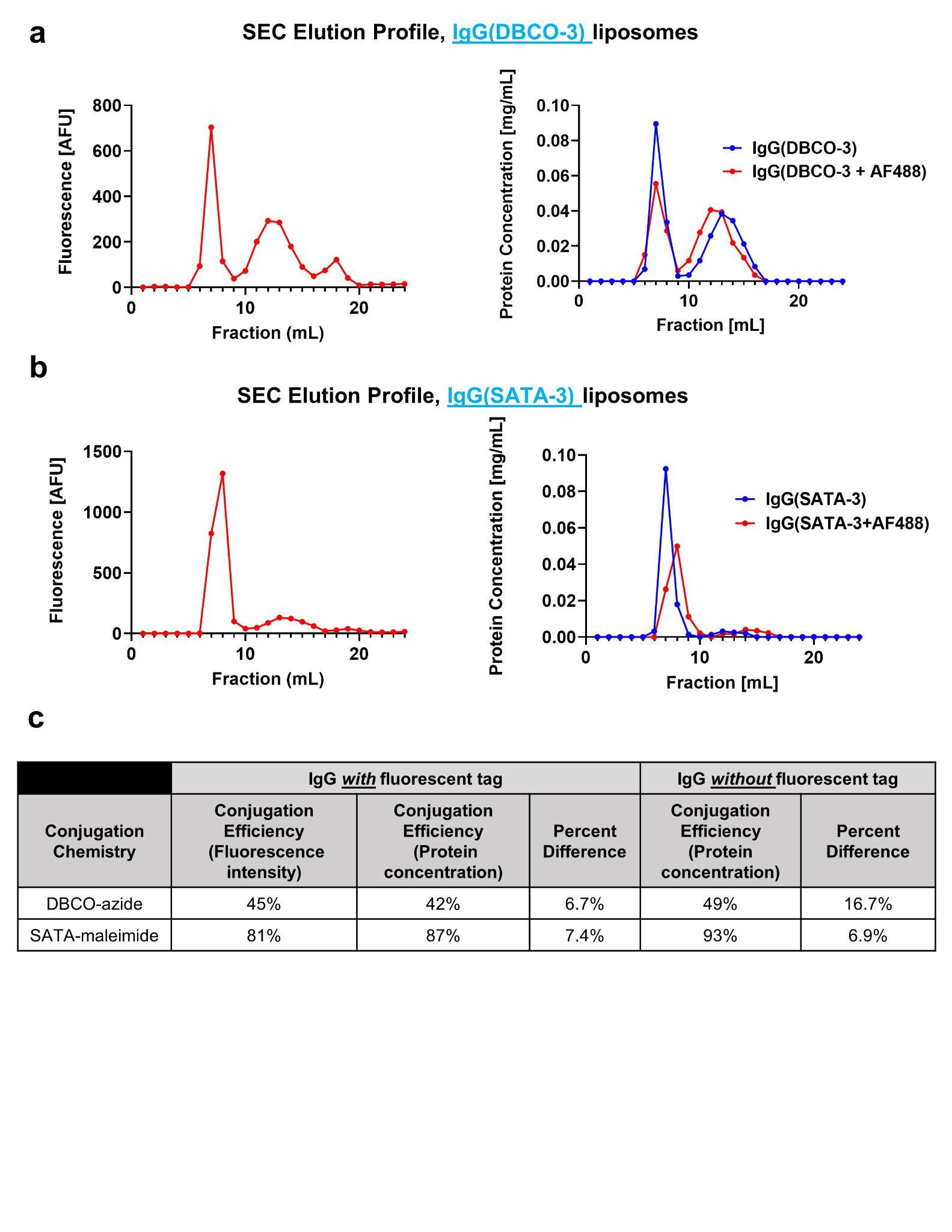


**Supplemental Figure S5. Modification of IgG with fluorescent tags does not impact conjugation efficiency.**

Studies were performed to assess the impact of fluorescent tags on conjugation efficiency of IgG to liposomes. Parallel conjugations were performed with both fluorescence-tagged IgG (red) and untagged IgG (blue). After conjugation, the mixture was purified via SEC to separate IgG conjugated to liposomes from unconjugated IgG. The amount of IgG in each fraction of SEC eluent was measured either via fluorescence intensity (a and b, left panels) or Bradford assay (a and b, right panels). The study was performed for both DBCO-azide (a) and SATA-maleimide (b) chemistries. IgG conjugated to liposomes elute at fractions 6 to 8, and unconjugated IgG elute at fractions 10 to 16 is unconjugated IgG. Note, the fluorescence peak at fractions 17 to 20 is unconjugated fluorophore. (c) Conjugation efficiencies were calculated as the amount of IgG conjugated to liposomes (peak at fractions 6 to 8) divided by the total IgG amount (peak at fractions 6 to 8 and peak at fractions 10 to 16). The conjugation efficiency was unaffected by fluorescent tags. Quantification via Bradford produces similar results (within 10%) as quantification via fluorescence intensity. Also, quantification via Bradford produces similar results (within 20%) for both tagged and untagged antibodies.

**
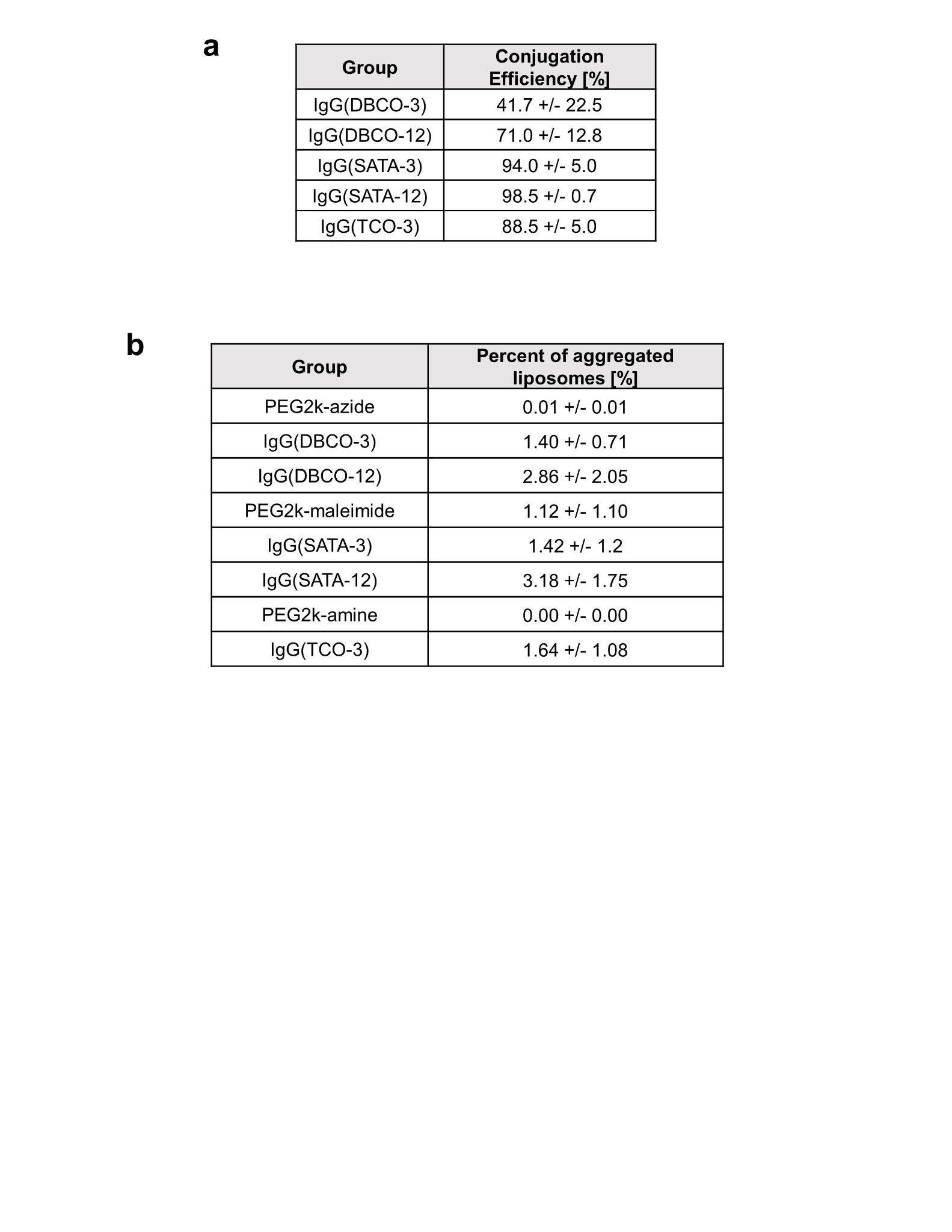
**

**Supplemental Figure S6. Conjugation efficiency and percent of aggregated liposomes for different conjugation chemistries.**

(a) The conjugation efficiency of IgG to liposomes is given for each conjugation chemistry. The efficiency varied across chemistries, and this difference was accounted for in order to achieve a final density of 200 IgG per liposome. See Supplemental Figures S3 and S4 for detailed description of the calculations. (b) The percentage of aggregated liposomes was calculated using data from NTA. Aggregated liposomes were defined as particles over 300 nm, chosen as double the size of PEGylated liposomes with no IgG conjugated (150nm x 2 for PEG-azide and PEG-maleimide liposomes). All preparations contained less than 4% aggregates. Increasing the modification of IgG (e.g., IgG(SATA-12) vs. IgG(SATA-3)) increases the percentage of aggregated liposomes. However, the percentage of aggregated liposomes was nearly identical when comparing SATA-maleimide and DBCO-azide chemistries.


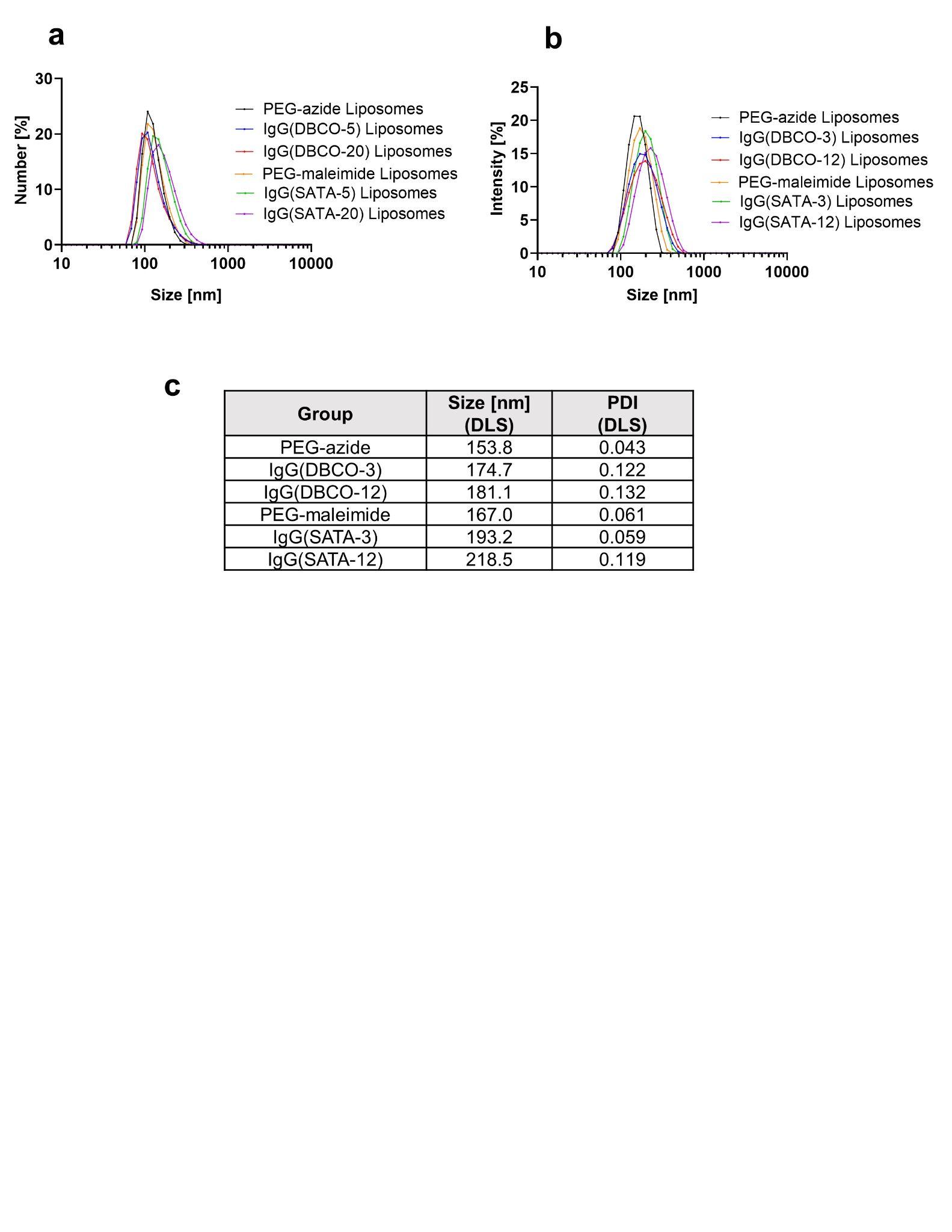


**Supplemental Figure S7. DLS characterization of IgG-liposome conjugates.**

Dynamic Light Scattering (DLS) data of antibody-liposome conjugates made using different conjugation chemsties. (a) Representative number-based size-distribution for all conjugation chemistries. (b) Representative intensity-based size-distributions for all conjugation chemistries. Note that intensity-based size distribution data exaggerates the presence of large-sized particles, because Rayleigh scattering intensity scales as size to the 6th power. (c) Table of Z-average and Polydispersity Index (PDI) for each condition. The results follow a similar trend seen with NTA analysis (Figure 1d).


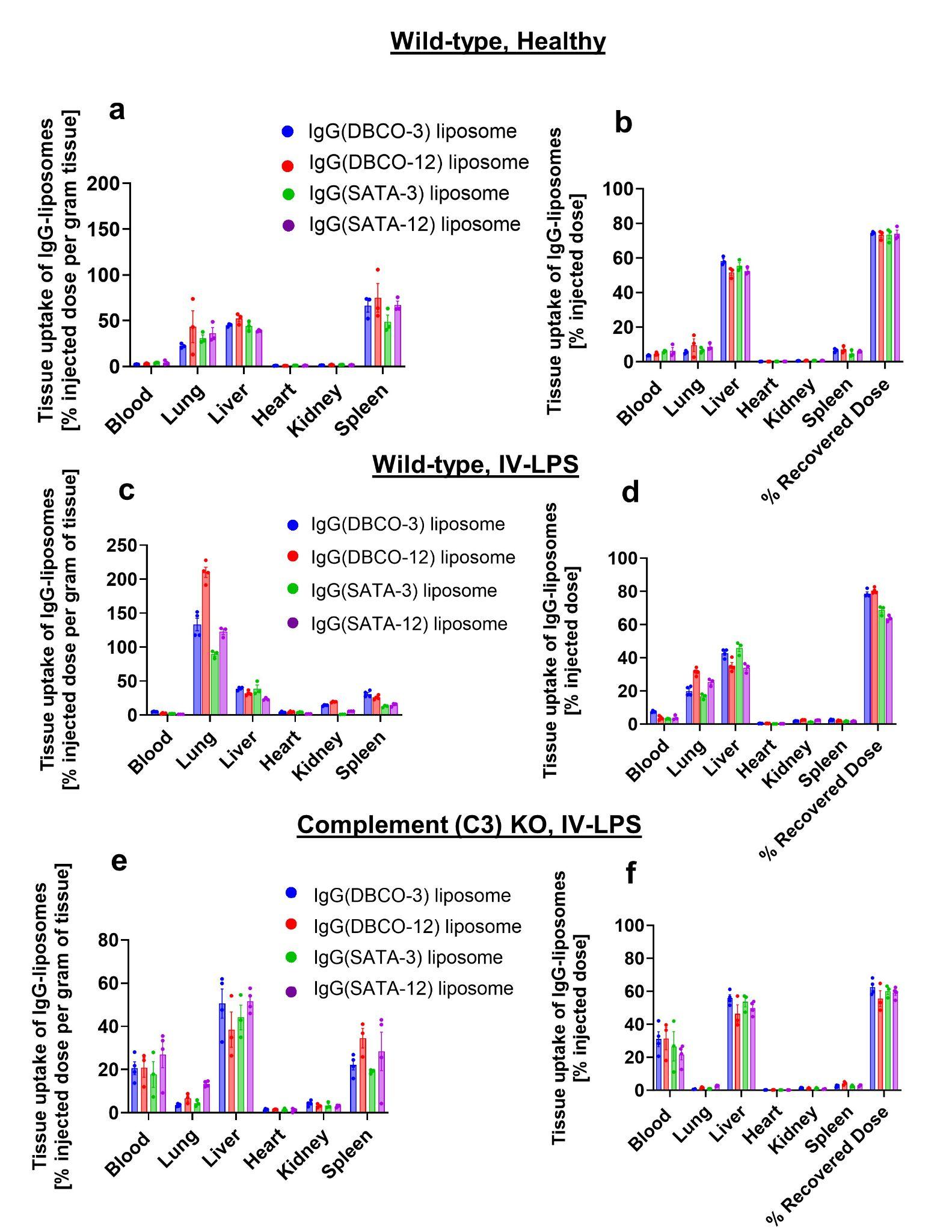


**Supplemental Figure S8. Complete biodistribution data for IgG-liposome conjugates,** as presented in Figure 2b to d. Data is presented for three different animal models: (a and b) Wild-type, healthy mice, (c and d) wild-type mice, with acute inflammation induced by intravenous bacterial lipopolysaccharide (LPS), and (e and f) mice with a knock-out of complement protein C3 (C3-KO) and with acute inflammation induced by intravenous bacterial LPS. Data is presented in two formats: (a, c, and e) percent of injected dose per gram of tissue and (b, d, and f) percent of injected dose per tissue.


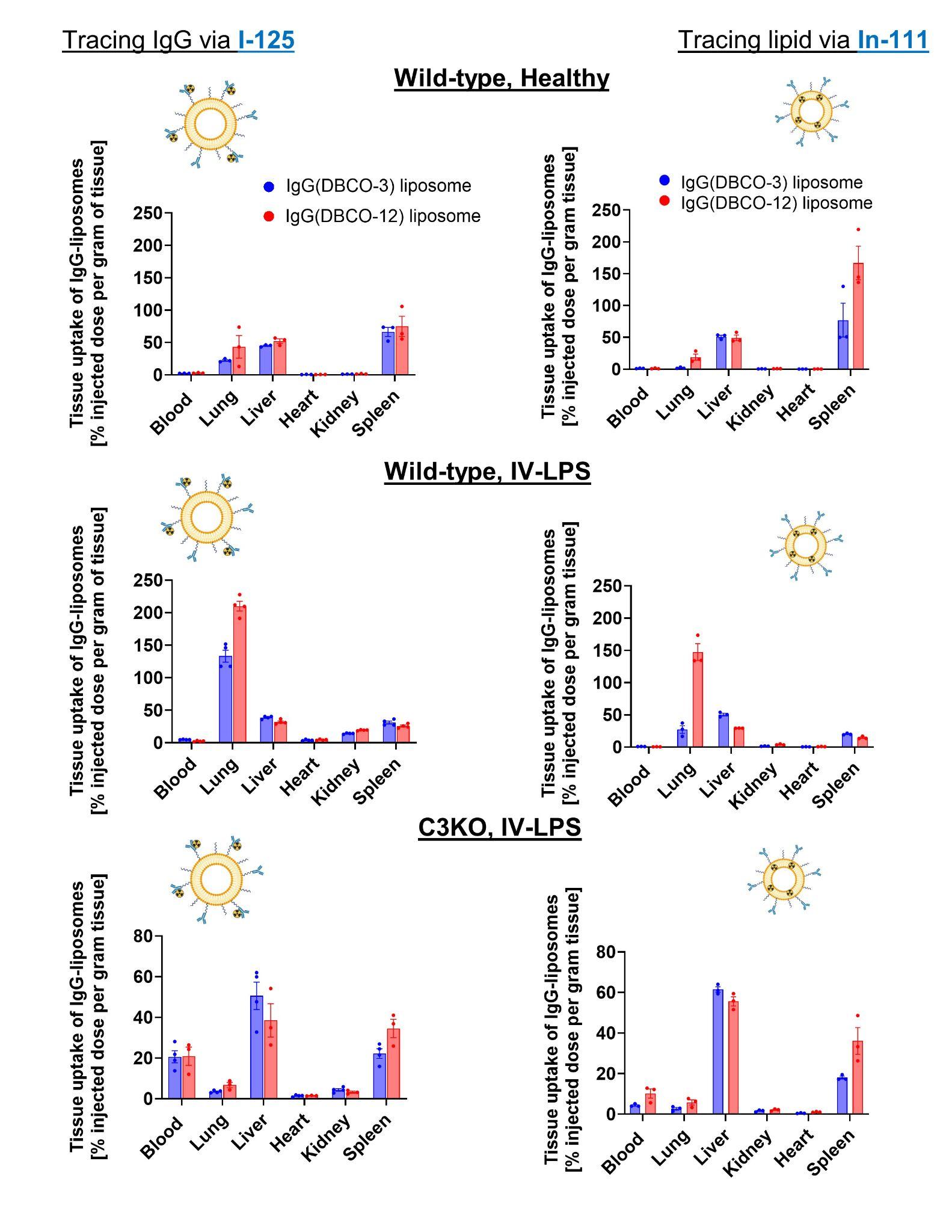


**Supplemental Figure S9. Control biodistribution using different radiotracing methods.**

Biodistribution studies were performed using both Iodine-125 (I-125) conjugated to IgG to trace the surface protein and Indium-111 (In-111) chelated to the liposome membrane to trace the membrane itself. Biodistribution studies were performed in healthy mice (top row), mice administered IV-LPS to induce systemic inflammation (middle row), and mice with knockout of complement protein C3 (C3-KO) also administered IV-LPS. With both methods, there is a significant increase in lung uptake in IV-LPS mice, and a complete elimination of lung uptake in C3-KO mice, demonstrating the necessary role of complement in inflammation-induced lung uptake.


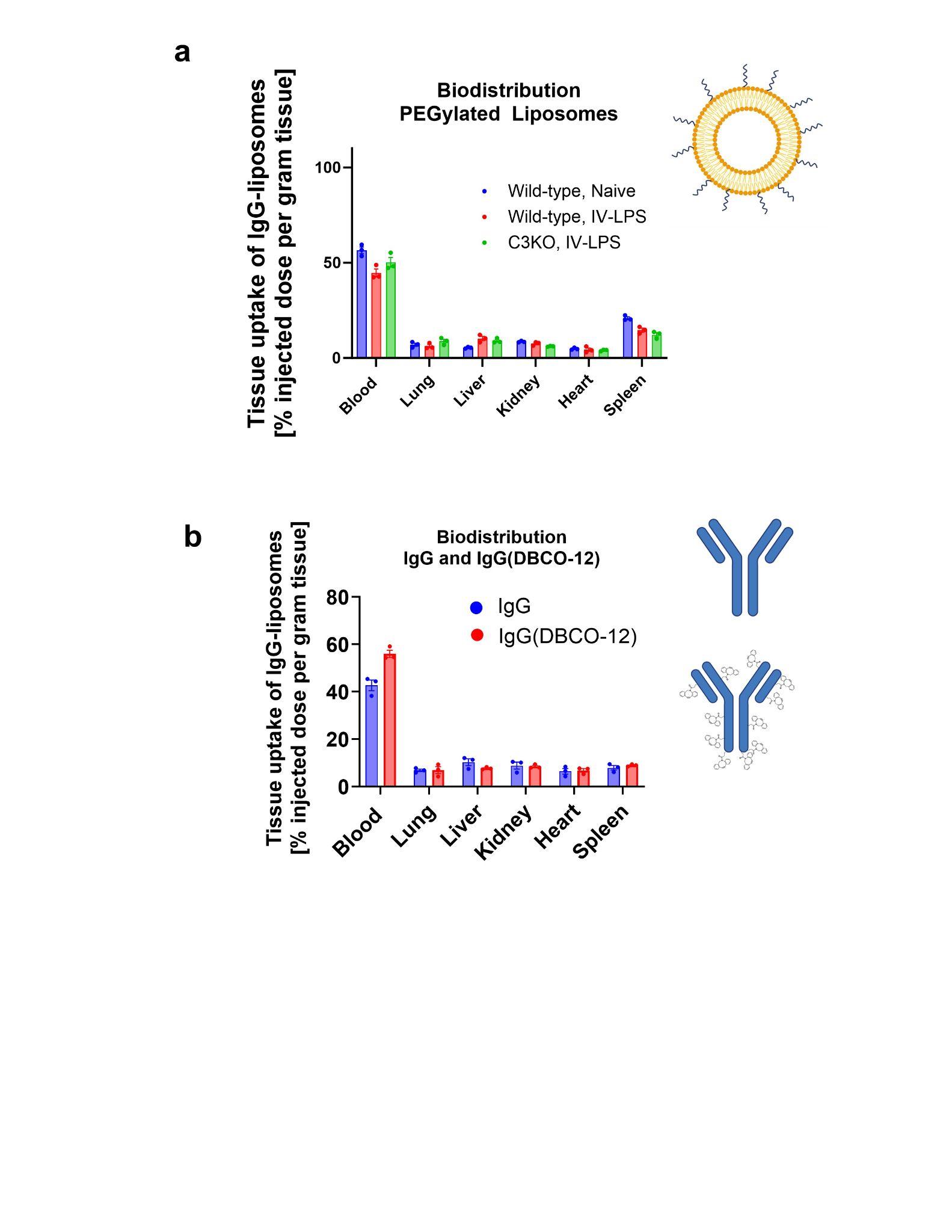


**Supplemental Figure S10. Biodistribution studies of individual components of antibody-liposome conjugates.**

Control biodistributions were performed on each component of the antibody-liposome conjugate separately.

(a) For plain liposomes (PEGylated, with no IgG conjugated), the liposome was traced via In-111 chelated to the lipid membrane. These data are also included in Figure 2b-d in the main manuscript. (b) IgG was traced via I-125 conjugated to the IgG. A condition with DBCO-modified IgG (IgG(DBCO-12)) was included to assess if DBCO-modified IgG is soluble after IV injection. All conditions largely remain in circulation for 30 minutes, with the majority of the dose in the blood and minimal distribution to tissue. This result aligns with literature reports of PEGylated liposomes and IgG, as both are known to be long-circulating. Together, these controls confirm that there is no significant lung uptake from the individual components of the antibody-liposome conjugates.


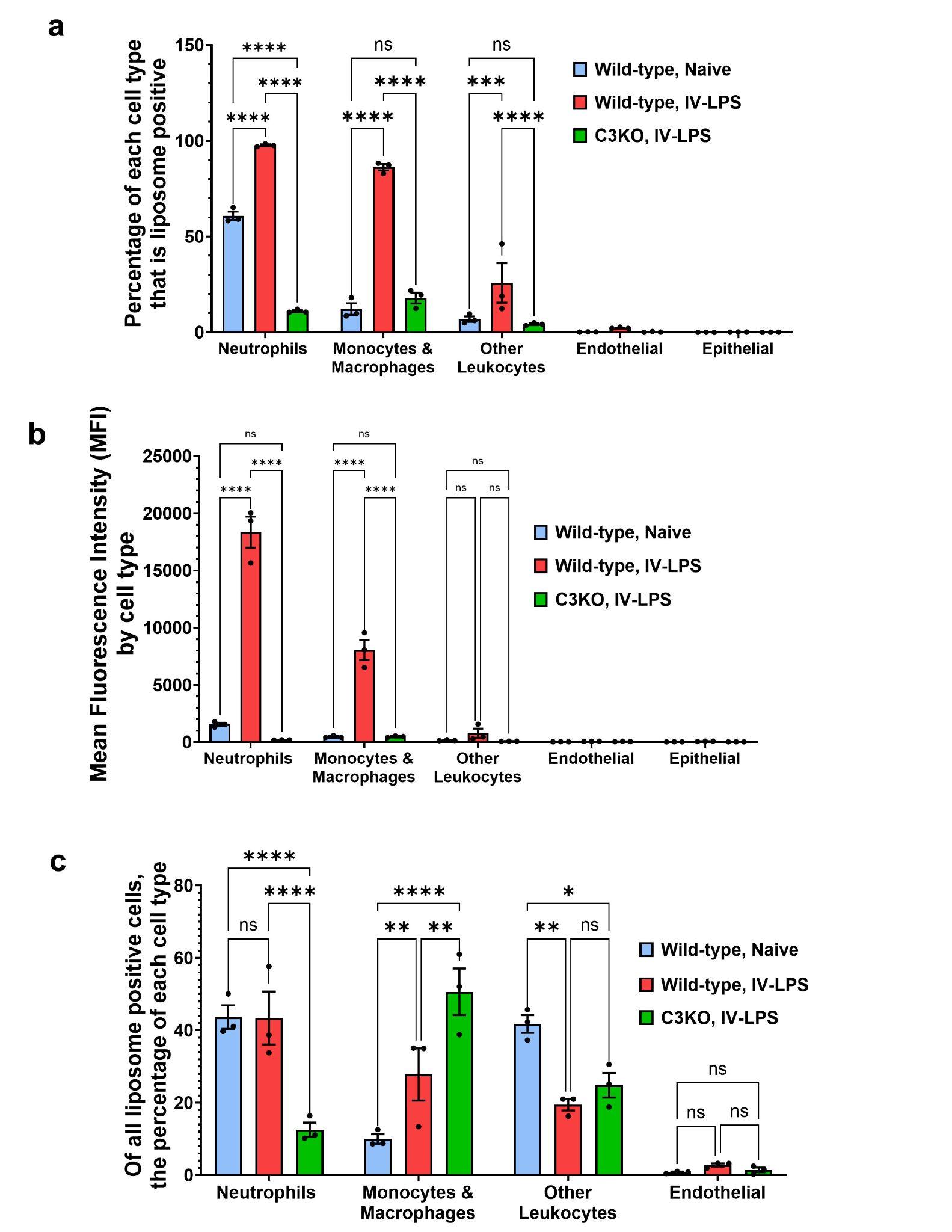


**Supplemental Figure S11. Cellular distribution of IgG-liposome conjugates.**

Flow cytometry studies were performed to assess the cellular uptake of IgG-liposome conjugates. IgG(DBO-3) liposomes were produced by conjugating 200 mouse IgG per liposome, and a fluorescent lipid was included in the formulation to facilitate flow cytometry analysis. Mice were IV-injected with IgG(DBCO-3) liposomes and 30 minutes later lungs were perfused, removed, and disaggregated to isolate single cells for analysis by flow cytometry. (a) Analysis of the percentage of each cell type that is liposome positive. The results indicate that neutrophils uptake liposomes more avidly than other cell types. In IV-LPS mice, nearly 100% of pulmonary neutrophils are liposome-positive (b) Mean Fluorescence Intensity (MFI) of all cell types. Neutrophils have the highest MFI of all cell types, in both healthy and IV-LPS mice, suggesting they take up the greatest quantity of liposomes.(c) Of all liposome-positive cells, the percentage from each cell type. Neutrophils are the most numerous liposome-positive cell type in both healthy and IV-LPS mice. In C3-KO mice, there is a dramatic decrease in cellular uptake, particularly neutrophil uptake.


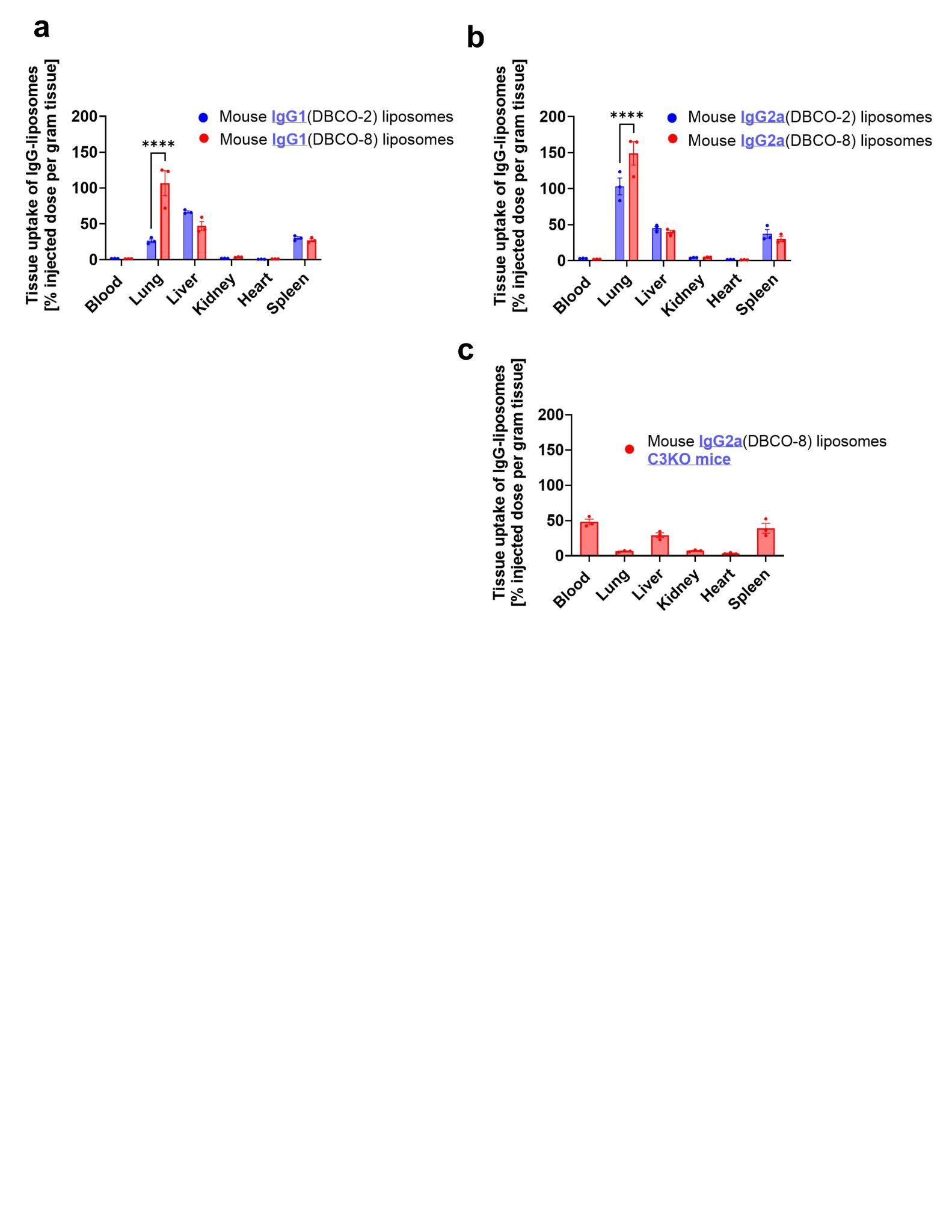


**Supplemental Figure S12. Biodistribution of IgG-liposome conjugates with distinct subclasses of murine IgG.**

The generalizability of complement-mediated lung uptake was assessed by performing biodistribution of IgG-liposome conjugated with monoclonal mouse IgG of multiple subtypes (IgG1 and IgG2a). Biodistributions were performed in IV-LPS mice (a and b) and C3-KO mice given IV-LPS (c). (a) Biodistribution of mouse IgG1-liposomes produced with DBCO chemistry, with varying amounts of DBCO per IgG. Increased modification with DBCO leads to a 4-fold increase in lung uptake. (b) Biodistribution of mouse IgG2a-liposomes. Again, increased modification with DBCO leads to a significant increase in lung uptake. For both conjugation chemistries, IgG2a has a greater lung uptake than IgG1, in agreement with literature that mouse IgG2a fixes complement more strongly than mouse IgG1. Biodistribution of the condition with the greatest lung-uptake (mouse IgG2a(DBCO-8)) in C3-KO mice shows no lung uptake, confirming the necessity of complement activation.


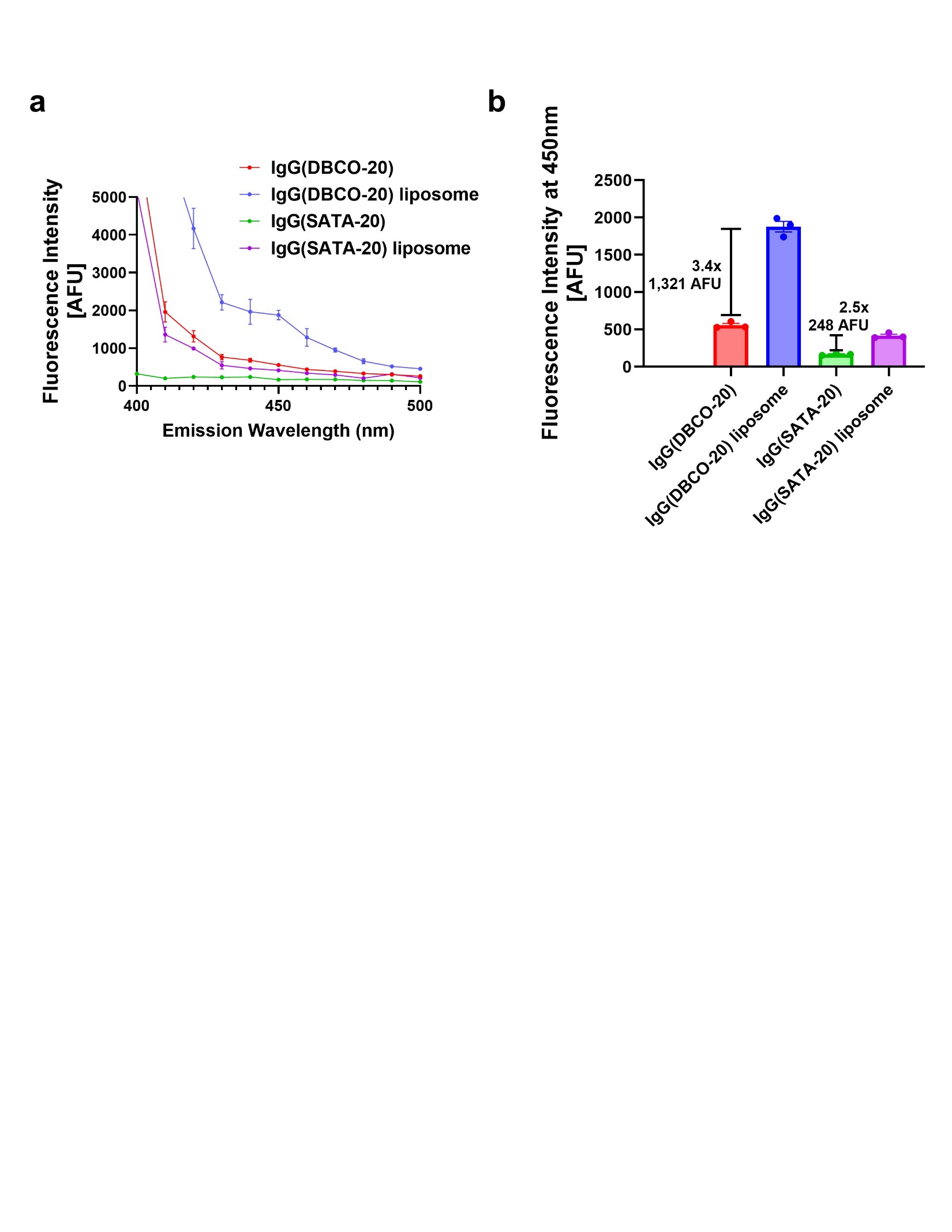


**Supplemental Figure S13. Measuring surface aggregation of IgG on liposomes using the aggregation-sensitive dye** **8-anilino-1-naphthalenesulfonic acid (ANSA).**

The aggregation-sensitive dye ANSA is used as an orthogonal method for measuring IgG aggregation. ANSA was added to IgG or IgG-liposomes, made using either DBCO-azide or SATA-maleimide chemistry. The total IgG concentration was equal for all conditions. (a) Emission spectrum of ANSA added to different IgG or liposome preparations. (b) Fluorescence emission intensity at 450nm. ANSA fluorescence increases for IgG-liposomes compared to IgG alone. This increase is greater for DBCO-azide chemistry, indicating the increased accessibility of hydrophobic domains when IgG(DBCO-12) is conjugated to liposomes.
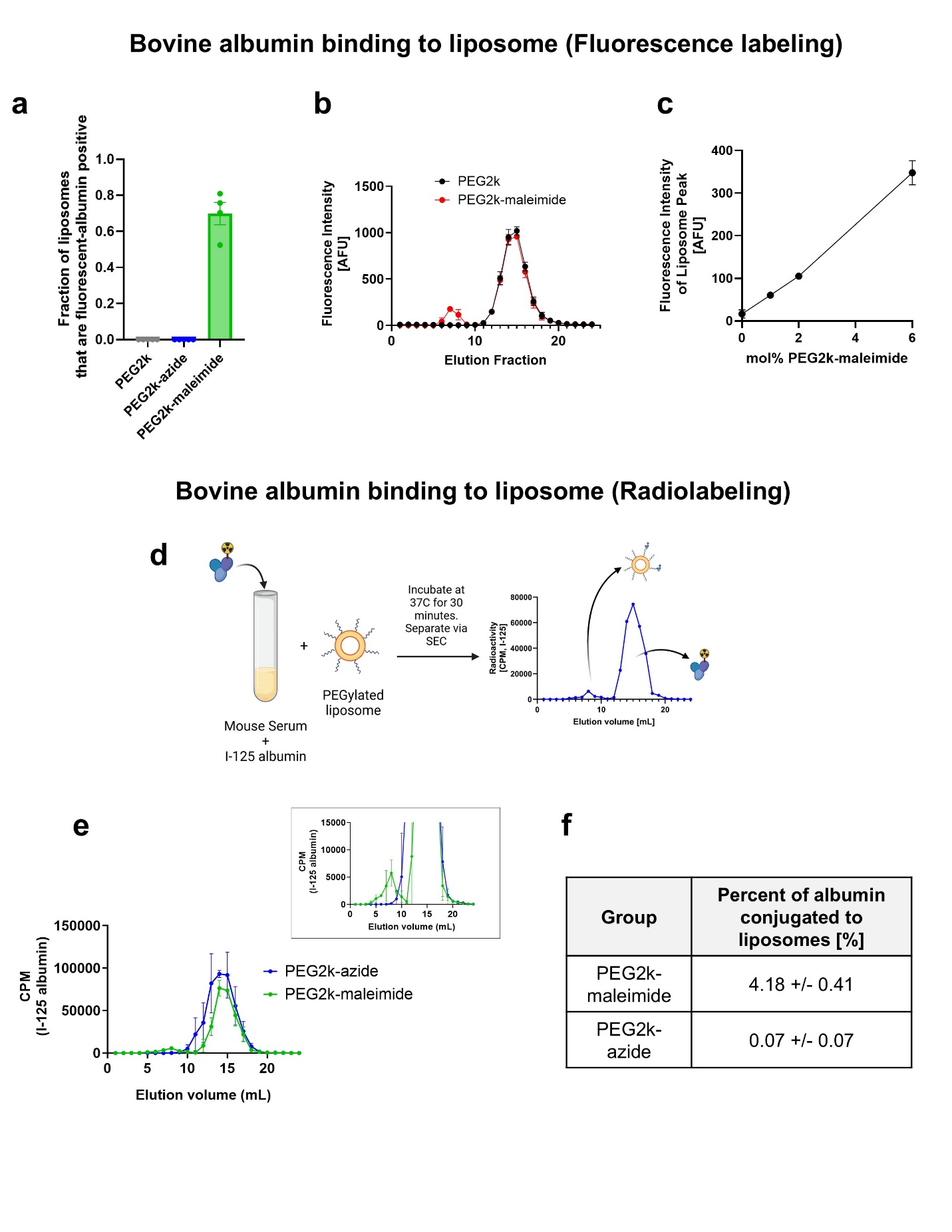


**Supplemental Figure S14. Bovine serum albumin (BSA) accumulates on liposomes containing maleimide functional groups but not liposomes containing azide functional groups.**

The binding of BSA to liposomes with either azide or maleimide groups was studied.

(a) Liposomes were incubated with fluorophore-labeled BSA, purified by SEC, and then the fraction of fluorescent-positive liposomes was measured by NTA. Liposomes with non-functionalized PEG (PEG2k) or azide-functionalized (PEG2k-azide) had no accumulation of albumin. Maleimide-functionalized liposomes were 70% positive for fluorescent albumin. (b) The elution profile of SEC purification after incubation of liposomes with fluorophore-labeled albumin. Liposomes elute in fractions 5 to 8, and the peak in fluorescence signal in these fractions indicate albumin binding to maleimide-containing liposomes. No fluorescence is present in fractions 5 to 8 of the negative control - PEGylated liposomes with no reactive group. The peak in fractions 11 to 19 is unconjugated albumin. (c) Liposomes were produced with varying amounts of DSPE-PEG2k-maleimide, ranging from 0 mol% to 6 mol%. DSPE-PEG2k with no reactive group was added in to maintain a total of 6 mol% PEGylated lipid. The total fluorescent intensity of the liposome peak from SEC elution (fractions 5 to 8) was summed. Increasing the mol% of PEG2k-maleimide results in increased binding of fluorescent albumin to liposomes, indicating that maleimide is driving albumin accumulation. (d) Graphic of method used to study albumin binding to liposomes in serum. BSA was radiolabeled with iodine-125 and added to fresh mouse serum. Liposomes were added to the serum/albumin mixture, incubated, and purified by SEC. (e) Elution profile of SEC purification, with radioactivity in each fraction measured. The inset shows a peak in fractions 5 to 9, indicating albumin binding to PEG2kmaleimide liposomes, even in the presence of serum proteins. (f) Quantification of the percent of albumin binding to liposomes, calculated by taking the area under curve (AUC) to the liposome peak divided by the total radioactivity.

**
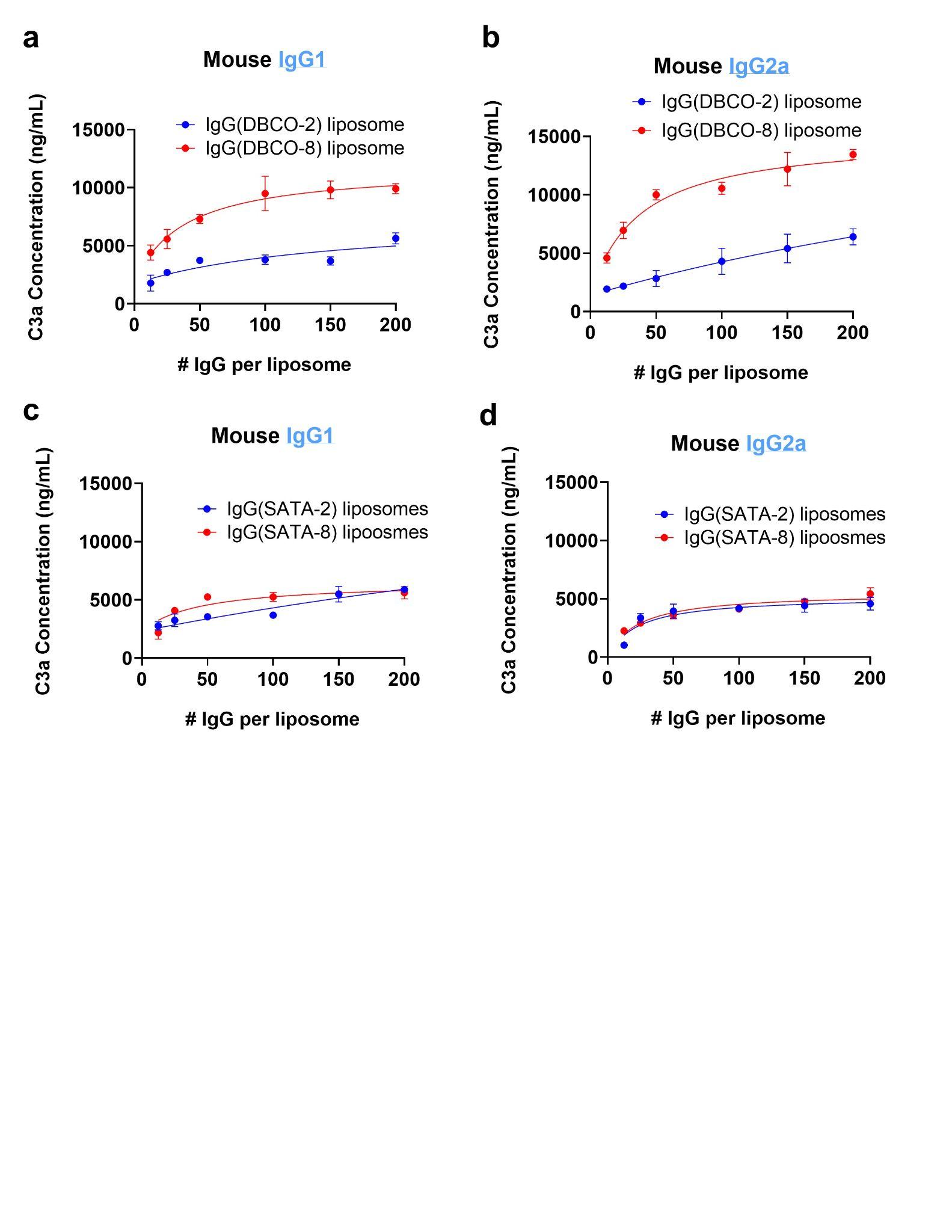
**

**Supplemental Figure S15. *In vitro* complement activation profiles of murine IgG of multiple subclasses.**

IgG-liposome conjugates, with varying amounts of IgG per liposome, were incubated with serum and C3a concentration was measured via ELISA. Studies were performed for both IgG1 (a and b) and IgG2a (c and d) subclasses. Increased modification of IgG with DBCO (8 vs. 2 DBCO per IgG) leads to significantly higher complement activation. However, increased modification with SATA has very little impact on complement activation. For SATA-maleimide chemistry, maximum complement activation is reached even at low IgG densities, supporting the hypothesis that the main driver of complement activation is free maleimide reactive groups on the surface of the liposome, as opposed to IgG conjugated to the liposome.


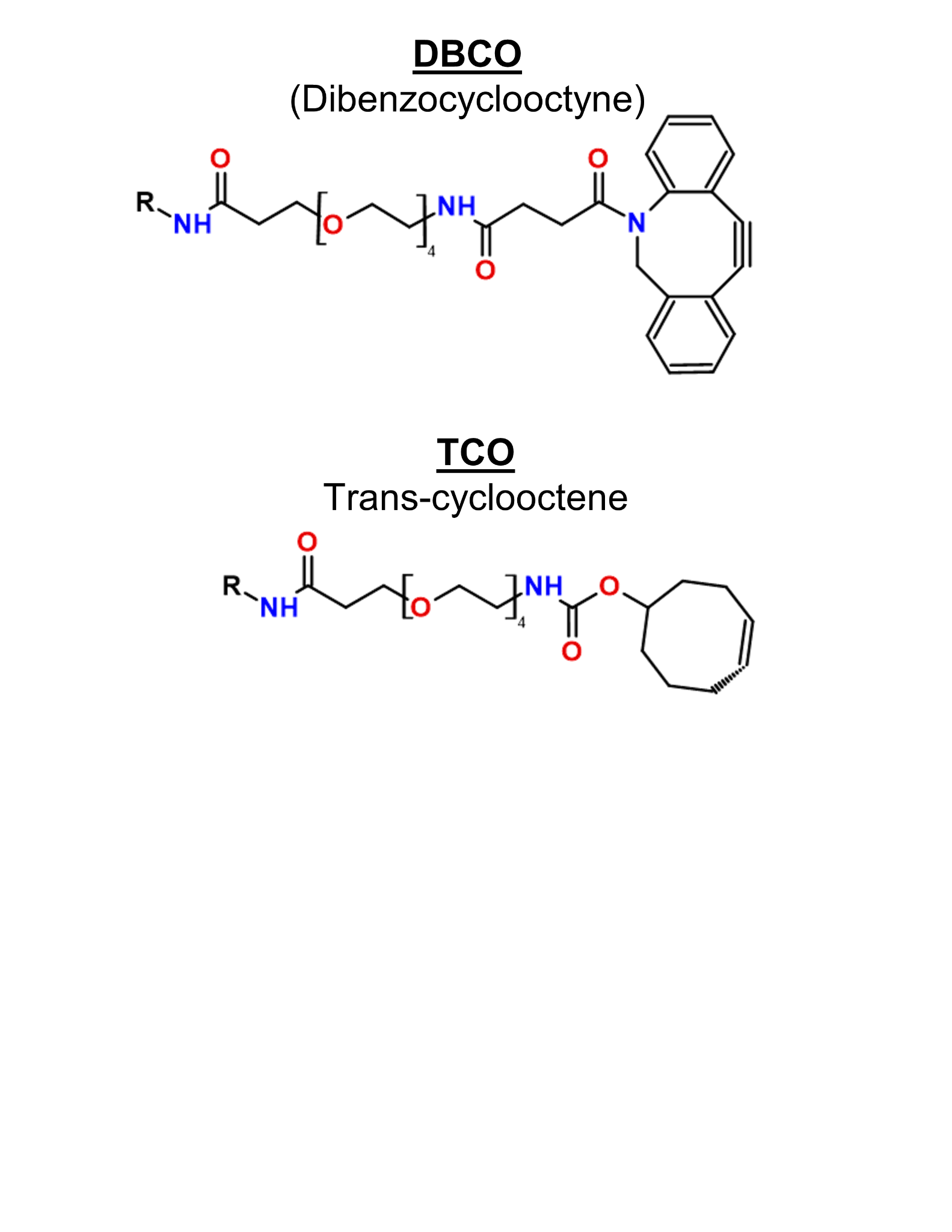


**Supplemental Figure S16.** Chemical structures for two click chemistries studied: DBCO (dibenzocyclooctyne) and TCO (trans-cyclooctene).


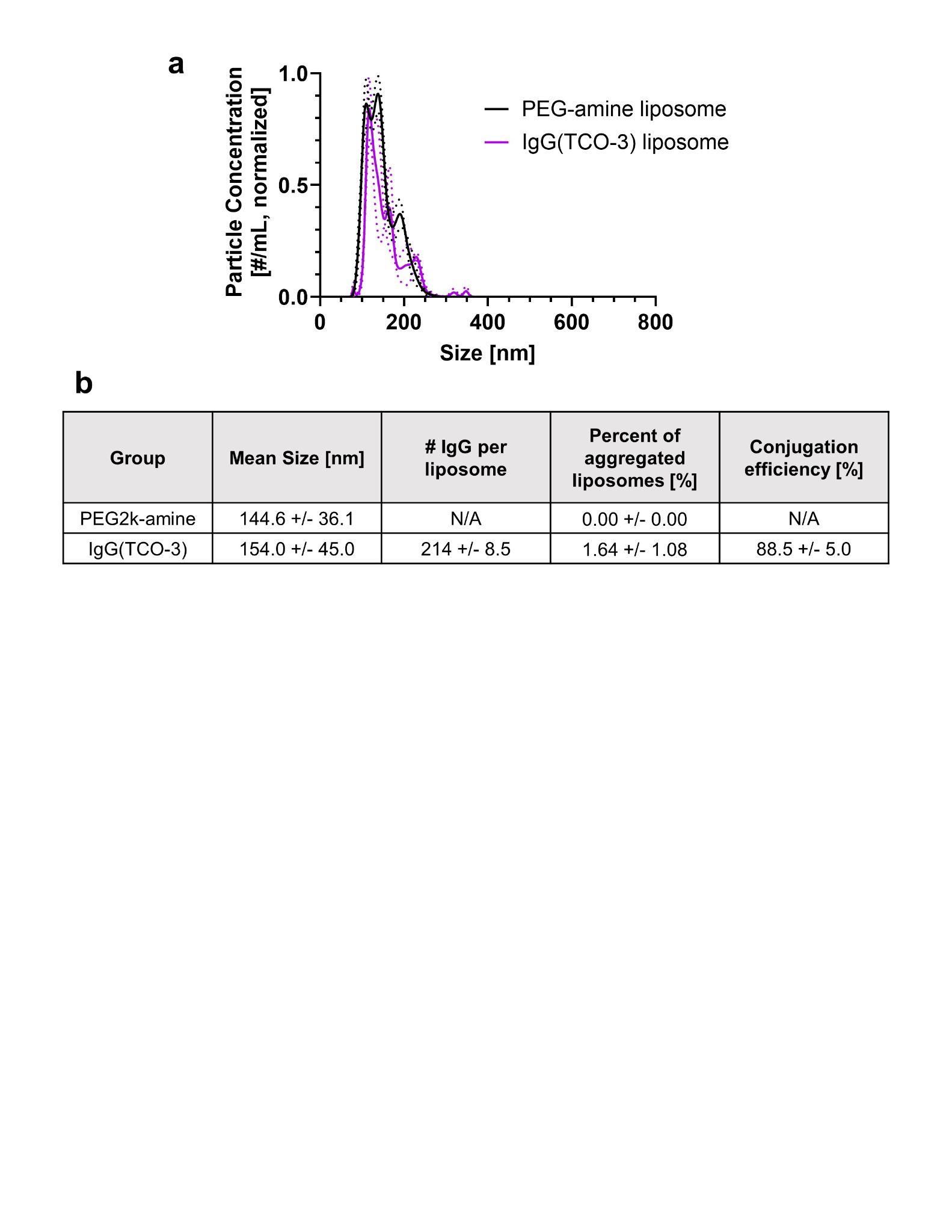


**Supplemental Figure S17. Characterization of IgG-liposome conjugates produced using TCO-tetrazine chemistry.**

(a) Nanoparticle Tracking Analysis (NTA) measurement of the size distribution of antibody-liposome conjugates. The size distribution of liposomes made using TCO-tetrazine chemistry is similar to the distributions of liposomes made using DBCO-azide and SATA-maleimide chemistries. (b) Additional characterization of antibody-liposome conjugates. Again, the results for TCO-tetrazine chemistry are similar to results using DBCO-azide and SATA-maleimide chemistries.


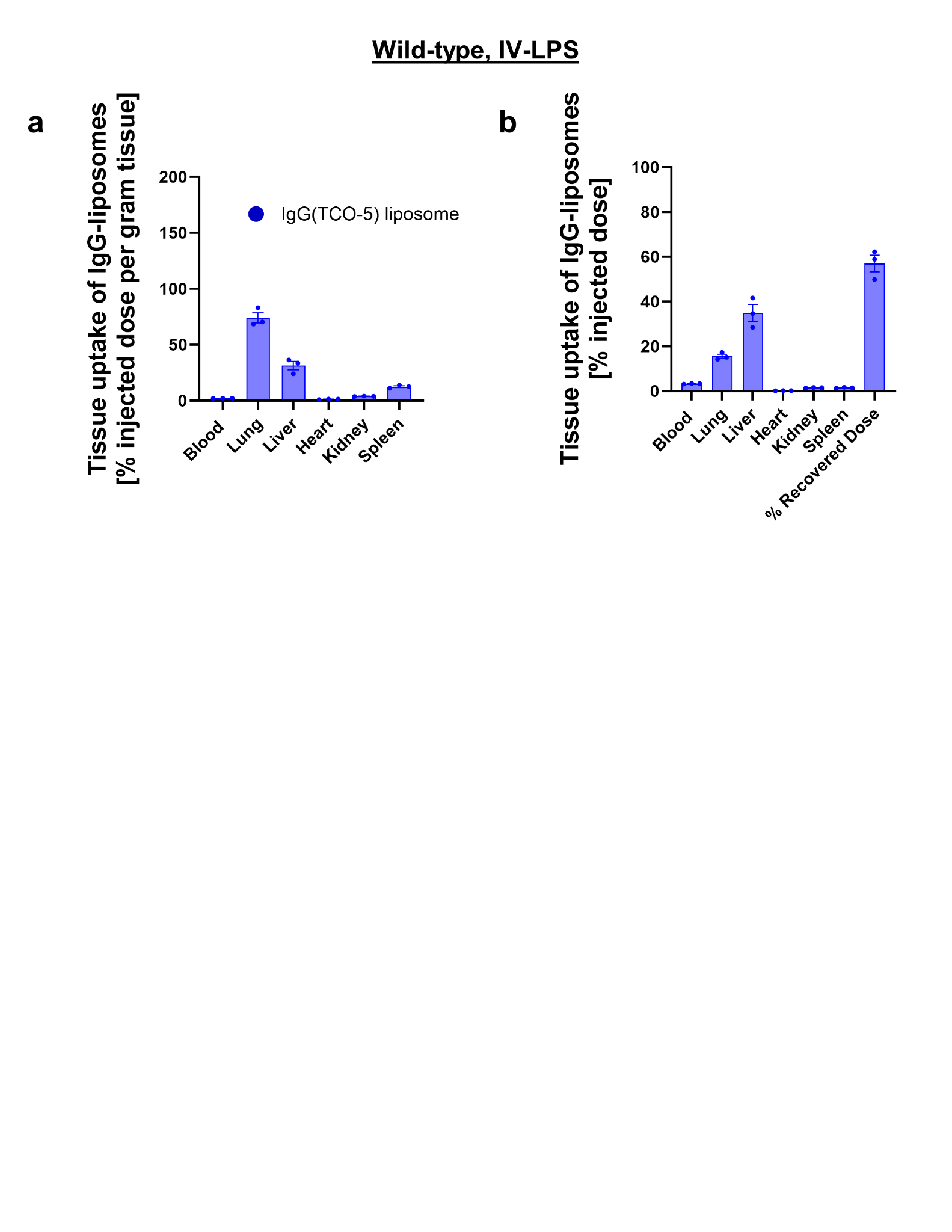


**Supplemental Figure S18.** **Complete biodistribution data for IgG-liposome conjugates using TCO-tetrazine conjugation chemistry.** Data is presented for wild-type mice, with acute inflammation induced by intravenous bacterial lipopolysaccharide (LPS). Data is presented in two formats: 1) percent of injected dose per gram of tissue and 2) percent of injected dose per tissue

References

(1) [Wiener, J.; Kokotek, D.; Rosowski, S.; Lickert, H.; Meier, M. Preparation of Single- and Double-Oligonucleotide Antibody Conjugates and Their Application for Protein Analytics. *Sci. Rep.* **2020**, *10* (1), 1457.](http://paperpile.com/b/6y1mkK/fwtcP)
